# A low-cost vibration isolation chamber – Making high precision experiments accessible

**DOI:** 10.1016/j.ohx.2022.e00264

**Published:** 2022-01-10

**Authors:** Håvard Vestad, Martin Steinert

**Affiliations:** Department of Mechanical and Industrial Engineering, Norwegian University of Science and Technology, Norway

**Keywords:** Vibration isolation, Sensitive experiments, Spring-damper, Prototyping

## Abstract

Mechanical vibrations greatly influence sensitive instruments and experiments, yet they are unavoidable. Commercial solutions that mitigate the transfer of mechanical vibrations into experiments and instruments are often associated with high prices and big footprints and are not readily available for low investment explorative testing, experimenting, and prototyping. In this paper, an open-source design for a vibration isolation chamber is presented that is constructed from readily available components and hardware such as off-the-shelf furniture and honey. An extensive guide on how to construct the simple spring-damper-based passive vibration isolation chamber is presented, and its performance is validated using a high-precision seismic accelerometer. The vibration isolation system consists of steel springs and dashpots made of steel spheres suspended in high viscosity honey. The system resonates at 1.2 Hz and successfully mitigates the transfer of vibrations of frequencies determined to be of critical interest in the 5–20 Hz range. The well-performing system has proven to be an invaluable asset in the laboratory toolbox when sensitive experiments are carried out and has already been used in a multitude of projects. The design is shared so that others may also benefit from this tool.

## Graphical abstract

**Figure:**
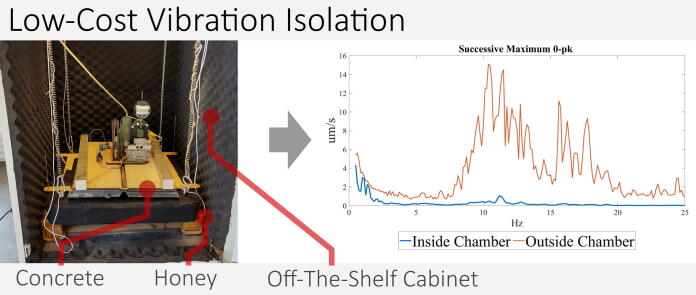

**Table:** 

Hardware name	*A Low-Cost Vibration Isolation Chamber – Making High Precision Experiments Accessible*
Subject area	• Engineering and materials science • Educational tools and open source alternatives to existing infrastructure • General
Hardware type	• Vibration Isolation • Laboratory Infrastructure • Experiment Setup
Closest commercial analog	*Passive vibration isolation optical tables*
Open source license	*CC by 4.0*
Cost of hardware	*400 USD*
Source file repository	doi.org/10.17605/OSF.IO/D6XAB

## Hardware in context

Through the progression of technological development and machining precision, we are increasingly able to observe, make, and work with nano-scale objects and phenomena. As the realm in which we work becomes smaller, the influence of mundane phenomena such as microseismical and microtremor vibrations become more significant, and when the dimensions of vibrations in some instances exceed the size of what we are working with, there is an obvious need for isolating specimens and machines from vibrations to produce credible results. This is especially important in modern microscopy, wherein reducing external vibrations is crucial for achieving high-resolution measurements [1], [2], but micro vibrations will also affect and potentially harm modern sensors [3] and sensitive machines [4], [5]. To this end, solutions are implemented, with a wide variety of concepts such as air springs [5], viscoelastic suspension [1], [6], active vibration detector - actuator systems [7], and negative stiffness systems [8]. Many of the solutions have commercially available alternatives, yet they are often associated with high costs. Both through their initial purchase but also due to large dimensions and high equipment weight, which in turn may translate to extensive transportation costs that need to be accounted for when browsing potential vibration isolation systems. For this reason, vibration isolation is not something that is commonly integrated into experiments and laboratories when the need is not explicitly identified. This makes vibration isolation a somewhat specialized laboratory feature, even though many experiments and prototype tests might benefit from simple vibration isolating measures.

**Fig. 1:**
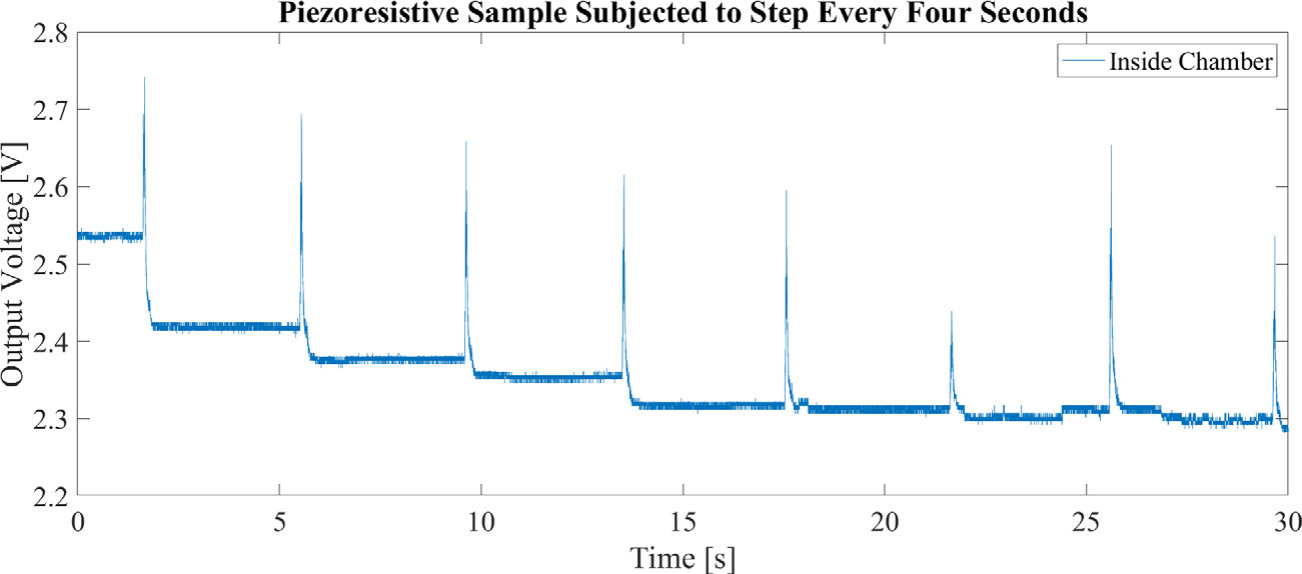
a) A piezoresistive material sample during a compression test. b) Illustrating a cyclic response to cyclic compression. c) Noise in the signal, hypothesized to be cause by vibrations.

One such benefit was discovered in our own makerspace-like prototyping laboratory, where the building in which it is located bridges over a trafficked road. Two bus routes that operate on the road utilize longer hinged busses that cause vibrations in the building between 5 and 20 Hz. Though not necessarily noticeable by humans, the vibrations were hypothesized to cause extensive noise in experiments in which piezoresistive composites were made and tested in the laboratory [9], [10]. To illustrate this, a small experiment was conducted. The data presented in Fig. 1 is the voltage measured at the output of a voltage divider formed by a resistor and a piezoresistive material sample. The sample was placed on a floor and slightly weighed down with a small wooden board. A metronome was used to time a heavy step on said floor, approximately 1 m from the sample every four seconds. Large voltage spikes, caused by increase in resistance in the material sample are evident following the steps. Further, the system settles on a slightly lower voltage following the induced vibration. With similar vibrations caused by less controllable sources, such as traffic, the presented vibration isolation chamber was constructed to further investigate piezoresistive materials under controlled conditions. The build was inspired by previous research in which purpose-built vibration isolation systems have shown excellent performances [6], [11], [12]. It has since been used in multiple experiments sensitive to vibrations and has been a beneficial addition to the laboratory toolbox.

## Hardware description

The presented vibration isolation system utilizes a simple passive spring-damper configuration built into an enclosure. The build is inspired by IKEA Hacking [13] and uses typical hardware that is readily available. The enclosure is based on an IKEA (Inter IKEA Systems B.V., Netherlands*)* kitchen cabinet that enables a fast build process and a small footprint that can be easily integrated into a laboratory setting. In addition, the enclosure provides some acoustic insulation as well as protection against drafts. Experiments can be observed from the outside through an observation window. The fiberboard construction of the cabinet makes it easy to add entry points for experiments with hand tools when needed. The main structures of the chamber can be easily disassembled and moved in pieces of less mass so that its placement in a laboratory and workshop may be changed quickly. The components used in the construction of the chamber were chosen with focus on availability and low price, to enable fast and low investment replication of the equipment.

### Design

**Fig. 2:**
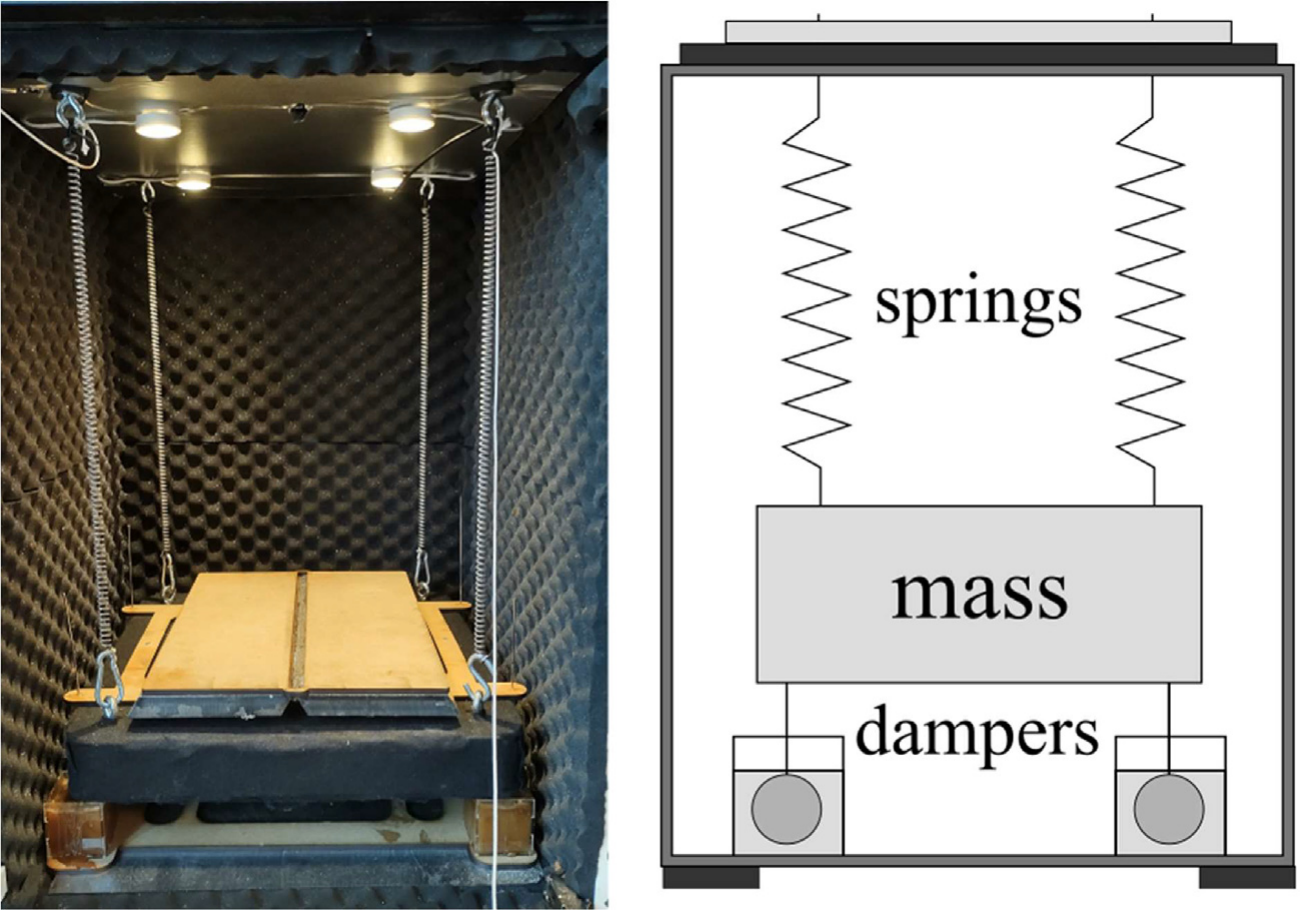
Four springs and viscous dampeners isolate the high mass table from external vibrations.

Passive vibration isolation can provide high performance and stability without the need for external power [4], [14]. The presented vibration isolation chamber uses four steel springs and four dashpot-like viscous dampeners, as illustrated in Fig. 2. The dampeners are made of steel spheres suspended in high viscosity honey. The damper design provides dampening in all degrees of freedom with minimal coupling between the directions [11]. For passive, linear, spring-damper vibration isolation systems, as the one presented, the absolute transmissibility of vibrations will peak at the systems resonance frequency. By increasing the damping in the system, the transmissibility at resonance decreases, but the absolute transmissibility for higher frequencies will in turn increase [15]; for this reason, systems are typically not designed overdamped, and rather some resonance at resonance frequencies are accepted [11]. The presented vibration isolation chamber targets reduction of frequencies associated with traffic at 5–20 Hz, has a resonance frequency of 1.2 Hz, and is underdamped.

#### Springs and table

The concrete table as presented in this build has a mass of 21.9 Kg. Additional mass is added to reach an operating mass of 46.3 Kg in the form of steel plates and/or equipment. Once operating mass is achieved, the springs are extended 0.2 m from their unstressed state to a total length of 0.48 m. Assuming even spring forces and Hookean behavior, the spring constant is estimated to be 0.57 N/mm.

Further for Hookean springs, the resonance frequency of a mass spring systems depends simply on its springs extension. Analytically, a resonance frequency can be estimated with equation 1, where *g* is gravitational acceleration, Δz is the spring extension, *m* is the mass of the table, and *k* is the spring constant, where for mass suspended from a Hookean spring *k* = *mg*/Δz. For a 0.2 m extension, the resonance frequency should be ∼ 1.11 Hz.

(1) $$f=\frac{1}{2\pi }\sqrt{\frac{k}{m}}=\frac{1}{2\pi }\sqrt{\frac{g}{\Delta z}}$$

**Fig. 3:**
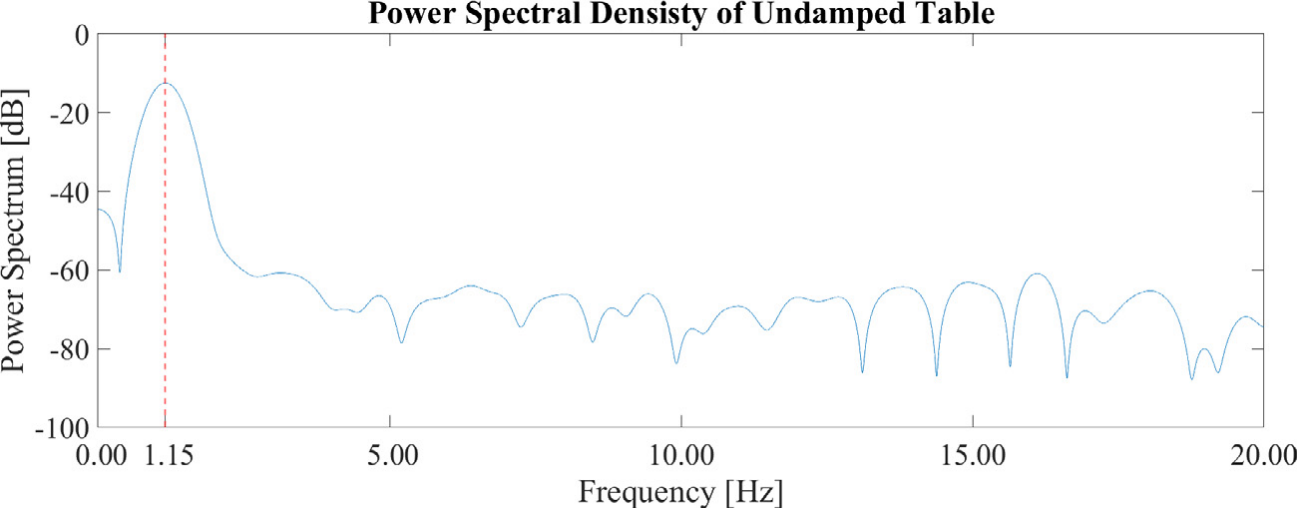
A frequency peak is apparent at 1.15 Hz for the power spectral density (PSD) analysis of the accelerometer data of the undamped table.

To gauge the resonance frequency of the table experimentally, the dampers were removed, and the table set into motion. A simple accelerometer app [16] was run on a smartphone (Moto G9 plus, Motorola) to collect accelerometer data. The data was analyzed in MATLAB (MATLAB ver. R2021a, Signal Processing Toolbox), subtracting its mean and running pspectrum(). The resulting data can be seen in Fig. 3. The actual resonance frequency is slightly higher than the calculated estimate, at ∼ 1.2 Hz. In the horizontal plane, a pendulum-system is formed where the pendulum arm length, *L*, is approximately the same as the total spring length. Its resonance frequency, *f,* is given by $f\approx \frac{1}{2\pi }\sqrt{\frac{g}{L}}$. Which for the presented table is lower than for the vertical system, at ∼ 0.7 Hz.

#### Damping

**Fig. 4:**
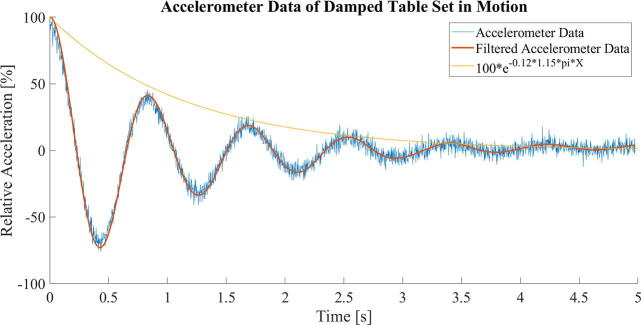
Accelerometer data of damped table. The yellow line visualizes the approximated damping ratio found through logarithmic decrement. (For interpretation of the references to colour in this figure legend, the reader is referred to the web version of this article.)

To achieve an under damped spring-damper system, four steel spheres, 15 mm in diameter, were attached to the table close to its corners. The spheres are submerged in high viscosity fluid in containers resting on the floor of the chamber. To achieve high viscosity at a low material cost with minimal degradation over time, it was decided to use honey. Honey is often considered Newtonian and of high viscosity, yet the former is not always the case. For honeys with high content of crystalline phases, the apparent viscosity is greatly increased yet non-Newtonian behaviors emerge [17]. The honey used in the presented system was chosen for its high viscosity due to high contents of crystalline phase. For this reason, accurately gauging its viscosity is not necessarily straight forward with simple resources. Rather, as a reference for reproduction, we present a simple investigation on the damping effect of the viscous damper design. As in 2.1.1, a simple accelerometer app [16] was run on a smartphone (Moto G9 plus, Motorola) to collect accelerometer data. The table was set into motion with the dampers installed. The accelerometer data is shifted by subtracting its mean and scaled to show the relative amplitudes as compared to the maximum value of the oscillation. The smooth() function in MATLAB is performed on the accelerometer data to apply locally estimated scatterplot smoothing (LOESS) with a span of 10%, as displayed in Fig. 4. By noting the first three positive and negative peaks, a logarithmic decrement is found by applying the natural logarithm on the value ratio of subsequent peaks. The mean value between the four ratios gives an estimated damping ratio of ∼ 0.12. A function describing the damped acceleration can be approximated as:

(2) $${A\times \sin(1.15\times 2\pi t+\theta )e}^{-0.12\times 1.15\pi t}$$

Where t is time in seconds, A is the initial amplitude, θ is the phase angle at t = 0, the resonance frequency of 1.15 Hz is inserted, and the estimated damping ratio of 0.12 is used. Further, estimates for the tables relative velocity and position during the sampled damping-sequence can be found using the cumtrapz() function in MATLAB, to confirm a damping ratio of ∼ 0.12 also for the positional data.

**Table:** 

**Characteristics**	
Spring constant	0.57 N/mm
Damping ratio	0.12
Extended spring lengths	480 mm
Spring extension	200 mm
Total operating mass	46.3 kg
Damper sphere diameter	15 mm
Resonance frequency (vertical)	1.2 Hz

## Design files

The vector files bellow are supplied so that the presented design and components can be replicated using a laser cutter or other computer numerical control (CNC) machinery. However, all components should be simple enough that sufficiently good design alternatives can be constructed using simpler methods and hand tools. Suggestions for design alternatives are provided in the build instructions.

**Table:** 

Design file name	File type	Open source license	Location of the file
*Concrete Table*	*dxf*	CC by 4.0	https://doi.org/10.17605/OSF.IO/D6XAB
*Door_Window*	dxf	CC by 4.0	https://doi.org/10.17605/OSF.IO/D6XAB
Acryilic_Damper_Tubs	dxf	CC by 4.0	https://doi.org/10.17605/OSF.IO/D6XAB
Damper_Sphere_Fastening	dxf	CC by 4.0	https://doi.org/10.17605/OSF.IO/D6XAB
Dampers_Baseplate	dxf	CC by 4.0	https://doi.org/10.17605/OSF.IO/D6XAB

**Concrete_Table.dxf:** This dxf file should be cut from a 6 mm medium density fiberboard (MDF) in a laser cutter to form a mold in which concrete is poured to create a table of high mass.

**Door_Window.dxf:** This dxf file is used to locate and cut an observation window into the chamber door and may be used to cut the window itself as well from 3 mm acrylic glass.

**Acryilic_Damper_Tubs.dxf:** This dxf file can be used to make fluid containers for the dashpot fluid dampers from 3 mm acrylic glass in the laser cutter. Alternatively, small containers can be sourced.

**Damper_Sphere_Fastening.dxf:** The spheres that interact with the dashpot dampers are attached to this plate which in turn attaches to the concrete table.

**Dampers_Baseplate.dxf:** The fluid containers for the dashpot dampers attach to this baseplate which is attached to the bottom of the chamber.

## Bill of materials

**Table:** 

**Designator**	**Component (Article Number)**	**Number**	**Cost per unit -USD**	**Total cost -USD**	**Source of materials**	**Material type**
Cabinet	METOD (502.056.26)	*1*	40,1	40,1	*IKEA (*Inter IKEA Systems B.V., Netherlands*)*	Non-specific
Hinges	UTRUSTA (404.017.84)	1	13,8	13,8	*IKEA (*Inter IKEA Systems B.V., Netherlands)	Metal
Door	VEDDINGE (202.054.30)	1	32,3	32,3	*IKEA (*Inter IKEA Systems B.V., Netherlands)	Organic
Top Plate	VEDDINGE (402.054.34)	1	25,1	25,1	*IKEA (*Inter IKEA Systems B.V., Netherlands)	Organic
Door Handle	BAGGANÄS (703.384.18)	1	8,3	8,3	*IKEA (*Inter IKEA Systems B.V., Netherlands)	Steel
Lights	HALVKLART (204.510.63)	1	11,9	11,9	*IKEA (*Inter IKEA Systems B.V., Netherlands)	Non-specific
Eye Bolts	M8 Eyebolts / hooks 100 mm (25–304)	2 (x4)	7,7	15,3	Hardware store Biltema(Biltema Sweden AB, Sweden)	Metal
Concrete Mix	Concrete mix (86–5584)	1 (25 kg)	6,6	6,6	Hardware store Biltema(Biltema Sweden AB, Sweden)	Composite
Magnet	Door closing magnets (88–366)	1 (×10)	4,8	4,8	Hardware store Biltema(Biltema Sweden AB, Sweden)	Metal
Gasket material	2 mm Rubber gasket sheet (60–241)	1	10,1	10,1	Hardware store Biltema(Biltema Sweden AB, Sweden)	Organic
Aluminum profiles	20×20mm×1000 Aluminum profile (208131)	2	6,2	12,5	Hardware Store ByggMAX (ByggMAX, Sweden)	Metal
Honey	Honey – High crystalline phase content	1 kg	10,8	10,8	Convenience Store	Organic
Bearing sphere	15 mm Bearing spheres	4	2,0	2,0	eBay (ebay.com)	Metal
Welding Rod	2 mm welding rod	1	15,0	15,0	Hardware store	Metal
Zip Ties	Zip Ties > 5 mm wide	16	4	4	Hardware store	Polymer
Wood Screws	25 mm Countersunk Wood Screws	18	5	5	Hardware store	Metal
Spring steel wire	Spring steel wire	12 m	40	40	Metal Supplier	Metal
MDF	610×1220×6mm Medium density fiberboard	3	12,0	12,0	Wood supplies store	Organic
Plexiglas	750×600×3mm Plexiglas Sheet (7392814100001)	1	22,2	22,2	Hardware store Coop Obs Bygg (Coop Norge SA, Norway)	Polymer
Acoustic Foam	Acoustic polyurethane foam (629375)	6	11,9	71,1	Hardware store Jula (Jula Norge AS, Norway)	Polymer
Foiled Acoustic foam	Acoustic foam (629378)	1	23,8	23,8	Hardware store Jula (Jula Norge AS, Norway)	Polymer
Felt	1×1m Felt	1	10	10	Hobby supply store	Non-specific
**Total Cost:**				396,7		

### Consumables

In addition to the bill of materials, we have included a list of consumables that have been used in smaller amounts to enable the build.

**Table:** 

**Designator**	**Component**	**Number**	**Source of materials**	**Material type**
CA glue	Cyanoacrylate (CA) glue	NA*	Hardware Store	Mono/Polymer
Wood Glue	Polyvinyl acetate wood glue	NA*	Hardware Store	Polymer
Spray Glue	Spray adhesive/contact glue	NA*	Hardware Store	Polymer
Duct Tape	Duct Tape	NA*	Hardware Store	Non-specific
Masking Tape	Masking Tape	NA*	Hardware Store	Non-specific
Elastic Bands	Elastic Bands	NA*	Hardware Store	Organic
Metal Wire	Any thin (0.5–1 mm) metal wire to help arm concrete	NA*	Hardware Store	Metal
Silicone Sealant	Silicone Sealant	NA*	Hardware Store	Polymer
CA glue Activator	CA glue Activator	NA*	Hardware Store	Non-specific
Solder	Solder for electronics	NA*	Hardware Store	Metal
Soldering Flux	Soldering Flux	NA*	Hardware Store	Non-specific
Putty	Modeling clay, blue tack, or vacuum bag sealing tape to seal holes when casting concrete	NA*	Hardware Store	Polymer
Acrylic glue	Acrifix 1S0116 (Evonik, Germany)	NA*	Hardware Store	Non-specific
*It is assumed that small amounts of the products are consumed and that the choice in product is of little significance to the end result; it is therefore recommended to use products already at hand and substitute when fit.				

### Tools

The following tools are used in this build, alternative designs that enable the use of different tools are discussed where appropriate:

• Drill driver with drill bits and screw bits
• Hammer
• Utility knife
• Pliers
• Hacksaw
• Soldering iron
• Spring winding tool
• Pen
• Measuring tape
• Multitool with metal grinding disk
• Concrete stirrer drill attachment
• Laser cutter
• Bucket
• Nitrile gloves
• Safety glasses

## Build instructions

### Chamber build

Start the build by assembling the chamber. The chamber provides a frame from which the vibration isolation system hangs and protects the vibration isolated table from acoustic noise and drafts.

#### Assemble base cabinet according to the instructions

**This step requires the following:** Cabinet, Drill driver with screw bits

**Fig. 5:**
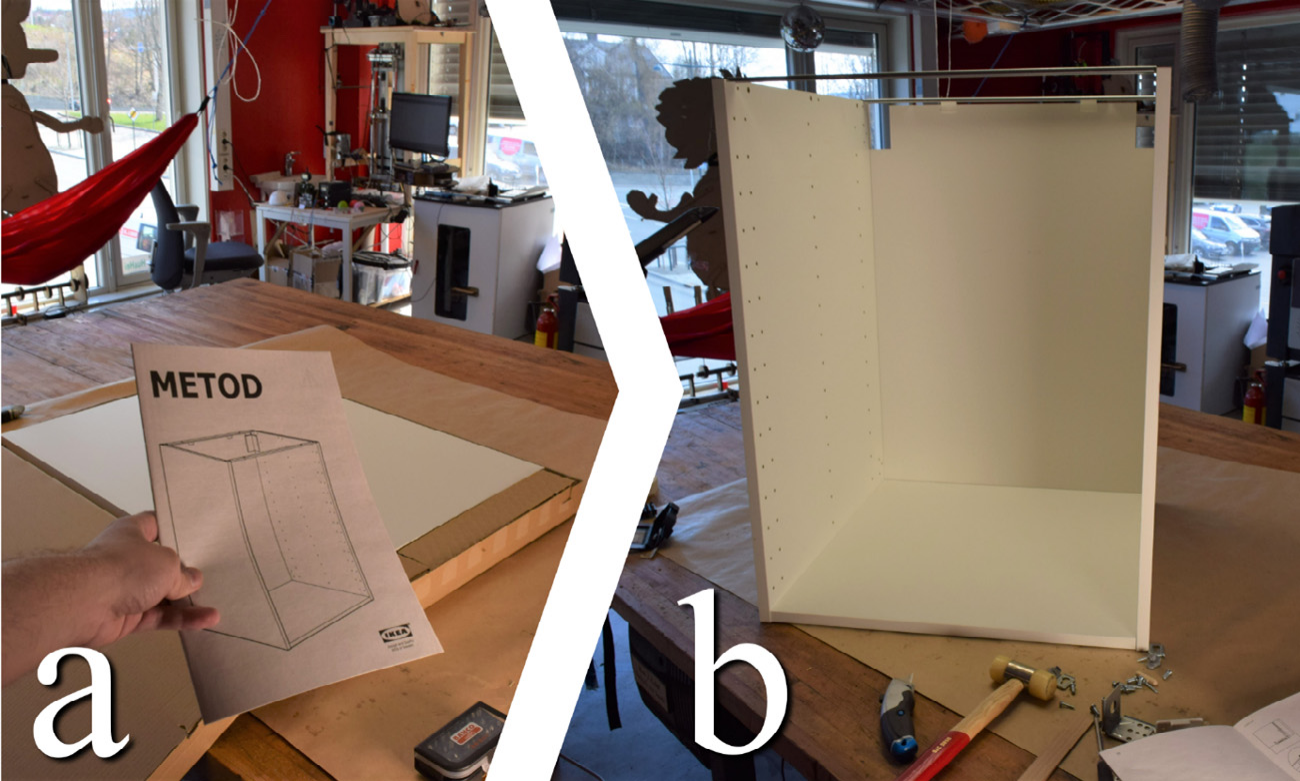
a) Instructions are included with the METHOD cabinet. b) Follow instructions to this stage.

The METOD cabinet should be assembled in accordance with its included instructions up until step 12. The assembled cabinet is shown in Fig. 5.

#### Add top plate

**This step requires the following:** Top Plate, 4×Wood Screws, Pliers, Drill driver with drill bits and screw bits, pen

**Fig. 6:**
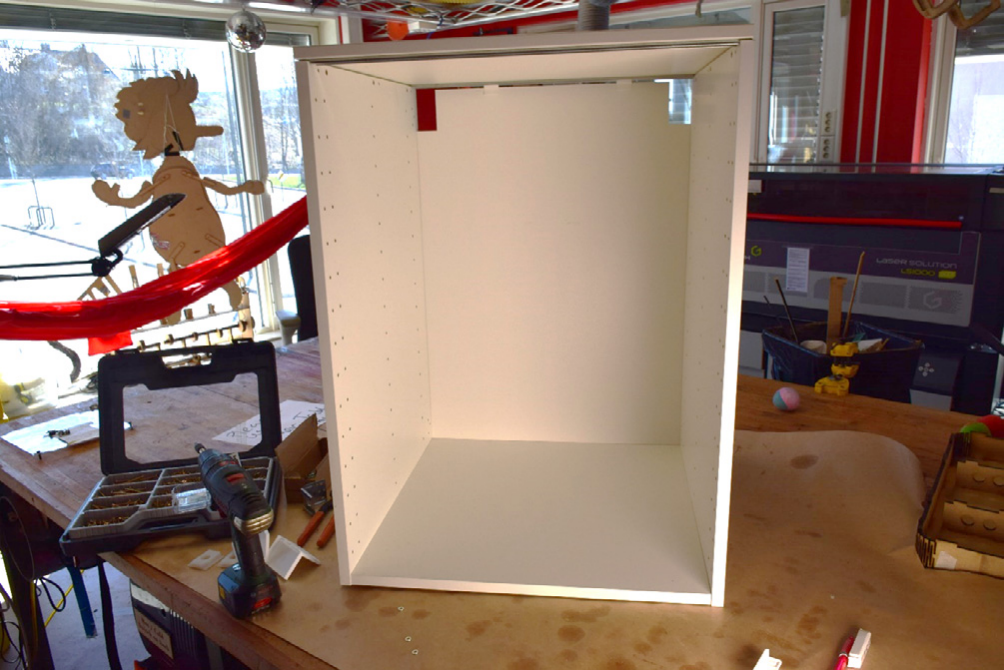
Top plate is placed on top of the cabinet and attached to the metal brackets with screws.

The steel brackets on top of the cabinet have four holes for fastening a benchtop. Lay the top plate on top and make sure that the front and sides are flush with the cabinet. Use a pen to mark the contour of the holes. Remove the plate and predrill holes with a 2 mm drill bit or another suitable size, depending on the screws used. Shorten the screws so that they are no longer than the thickness of the top plate by cutting them with pliers. Add the plate to the top and fix it with screws. The end of this step is illustrated in Fig. 6.

#### Add backing plate

**This step requires the following:** MDF, 12×Wood screws, Laser cutter or saw, Drill with drill bits and screw bits, Masking tape or duct tape

**Fig. 7:**
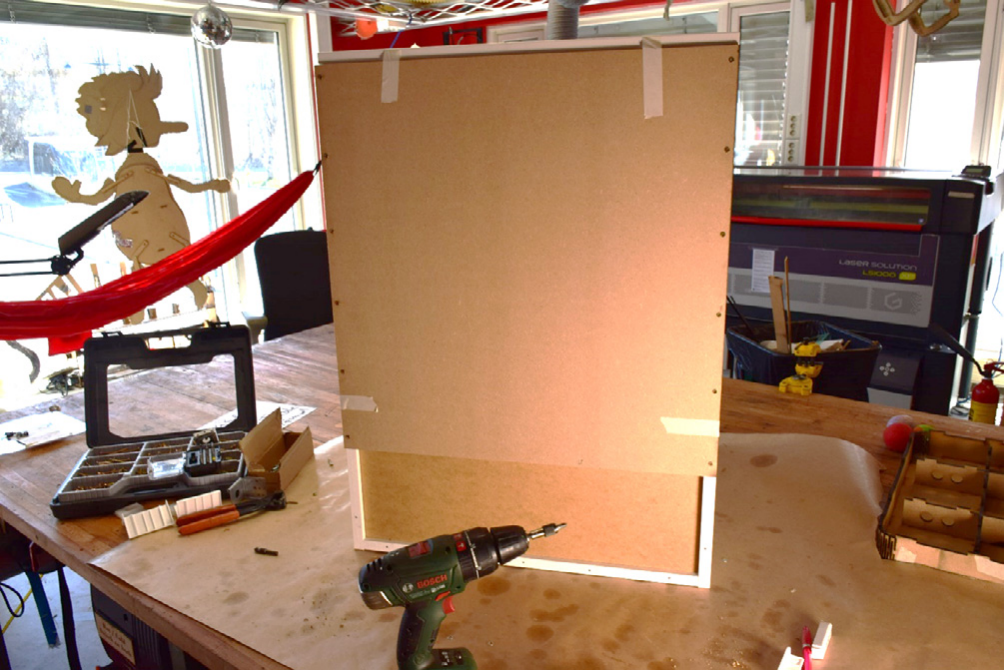
6 mm MDF plate is cut to 600×600mm and screwed into the walls on the back of the chamber.

The cabinet structure offers little lateral stability on its own. To counter this, we suggest adding a 600×600×6mm MDF plate to the back of the cabinet. The plate is screwed along the edges into the walls of the cabinet, supporting the structure against shear in the lateral direction, as seen in Fig. 7. Alternatively, diagonal supports and brackets may be added along the corners on the outside or inside of the chamber.

#### Cut holes for observation window

**This step requires the following:** Door_Window.dxf**,** Laser cutter (alternatively drill driver with drill bit and jigsaw), Utility knife, Masking tape, Hammer

Before mounting the door to the cabinet, a hole should be cut into its center to accommodate an observation window. For this, we utilized a laser cutter, centering a 550×230mm hole with rounded edges on the plate. The design file Door_Window contains a design for this hole, as well as a rectangle that represents the outer edges of the door. This should make it easy to center the hole on the door by referencing the laser cutter to the corner of the outer rectangle. If a laser cutter is not available, it is possible to cut the window hole by drilling the corners and sawing between them with a jigsaw.

If a laser cutter is used, we recommend using masking tape on and around the approximate area of the cut. This will prevent sooting of the door.

**Fig. 8:**
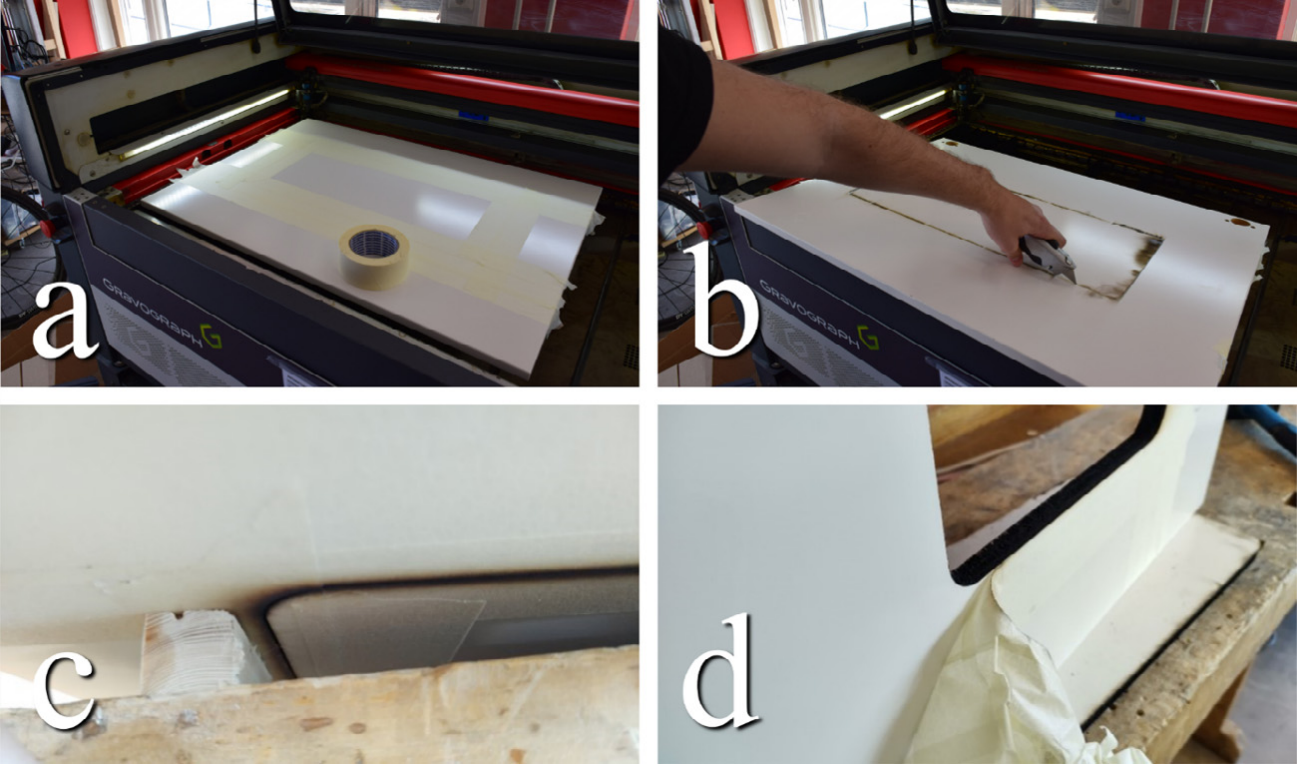
a) Mask of the approximate area of where the cut will happen with a wide border. b) Cut is traced by knife. c) Place a block along the edge of the cut before knocking it out with a hammer. d) Remove masking.

In our experience, the fiberboard will produce extensive sooting when cut with a laser. Depending on the machine, It might be challenging to get a clean cut along the contour and it will take multiple passes. When it is possible to make out the contour on the backside of the door, and the laser has started to penetrate the door in some areas, it is possible to finish the cut by hand, by tracing the contour with a utility knife and knocking the inner portion out with a hammer. This process is illustrated in Fig. 8.

#### Glue window

**This step requires the following:** Door_Window.dxf**,** Laser cutter (alternatively jigsaw), Acrylic glass, Silicone sealant, Nitrile gloves

**Fig. 9:**
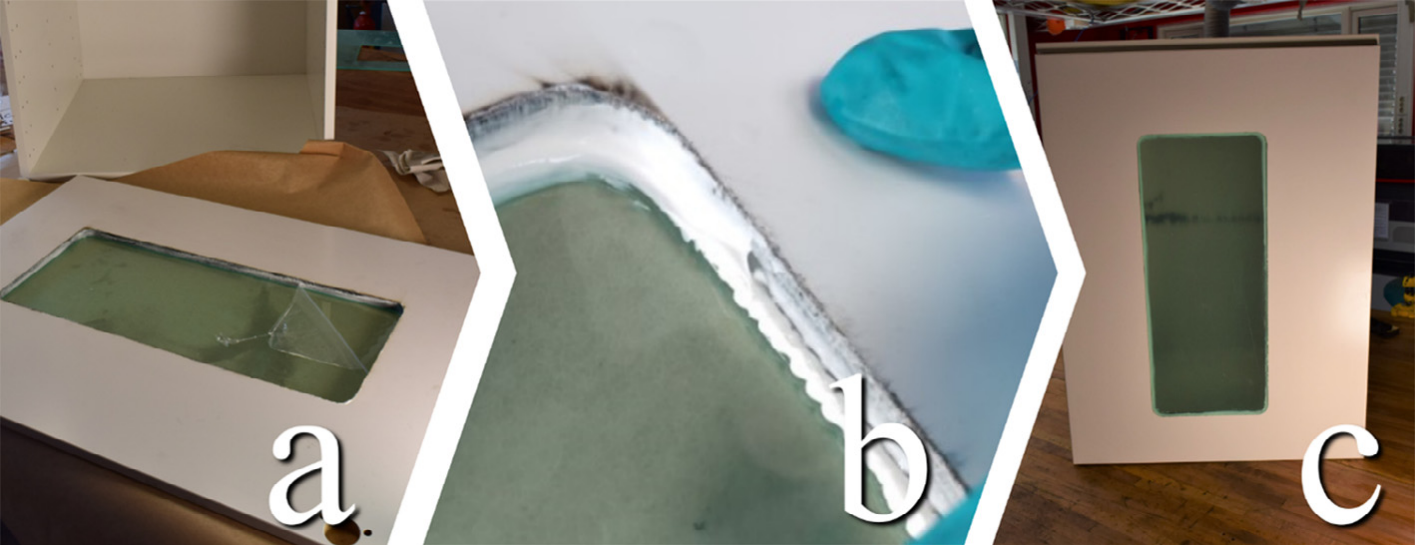
a) Remove the protective film. b) Apply glue along the edge and smooth out. c) Finished installing.

The window is cut from the same file, Door_Window.dxf, or dimensions as in the previous step from the 3 mm acrylic glass sheet. The finished cut is glued into the door, either using silicone or CA glue. The latter provides a less visible glue joint, whereas the silicone sealant seals any potential air movement and drafts between the bonding surfaces. Apply CA glue in small daps along the edge of the window or apply silicone sealant along the entire edge. The silicone sealant can be rounded by dragging a glowed finger wetted by soapy water or isopropyl alcohol along the edge, as illustrated in Fig. 9. Let cure.

#### Attach door

**This step requires the following:** Door, Hinges, Drill driver with screw bits

**Fig. 10:**
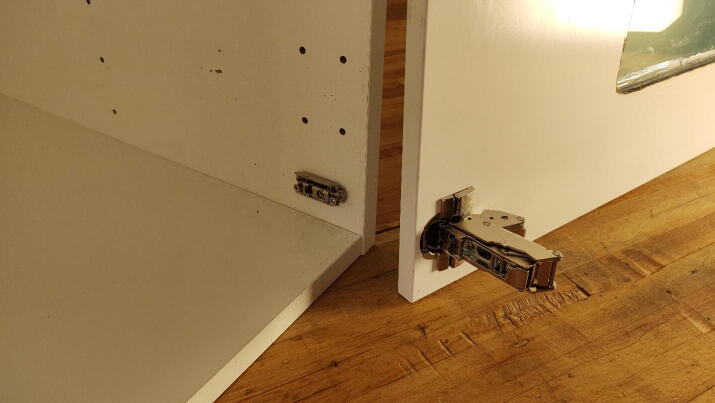
Hinges installed on door.

The hinges are easily installed by following the included instructions (Fig. 10). Make adjustments so that the door is centered and leveled with the frame of the cabinet.

#### Attach door handle

**This step requires the following:** Door Handle, Pen, Masking tape, Drill driver with screw bits and drill bits

**Fig. 11:**
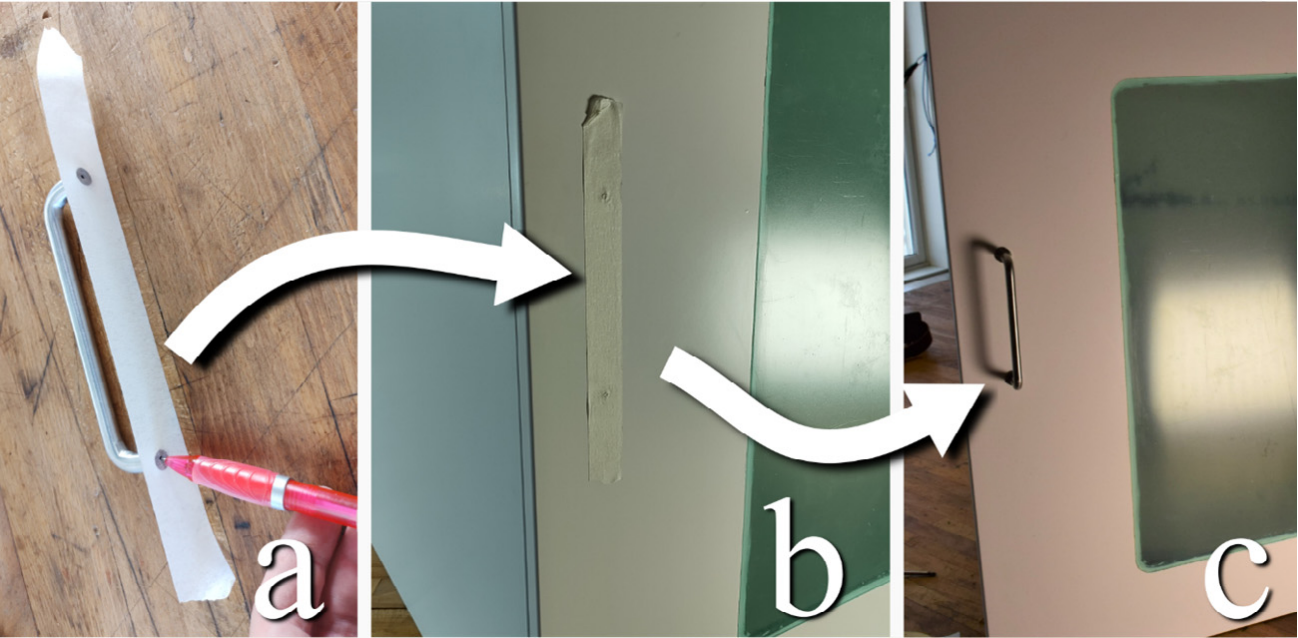
a) Mark hole pattern on a piece of masking tape. b) Place the tape in the desired position. c) Drill according to markings and install the handle.

The door handle is attached with screws. To space and place the holes correctly, a piece of masking tape can be attached to the base of the handle. Mark the holed with a pen. The tape is then placed in the desired location on the door. Drill holes on the markings and attach the handle with the included screws (Fig. 11).

#### Add acoustic foam to inside of chamber (optional)

**This step requires the following:** Acoustic foam, Foiled acoustic foam, Utility knife

The spring and dampening system will provide vibration isolation for lower frequencies. If higher frequencies are of concern, sound insulation of the chamber or the room into which the chamber is placed might be necessary. A thorough method for this is not provided in this build, as our primary concern is vibration in the range 5–20 Hz. However, the simple structure of the chamber provides surfaces on which it is possible to attach self-adhering polyurethane foam for some basic sound absorption.

**Fig. 12:**
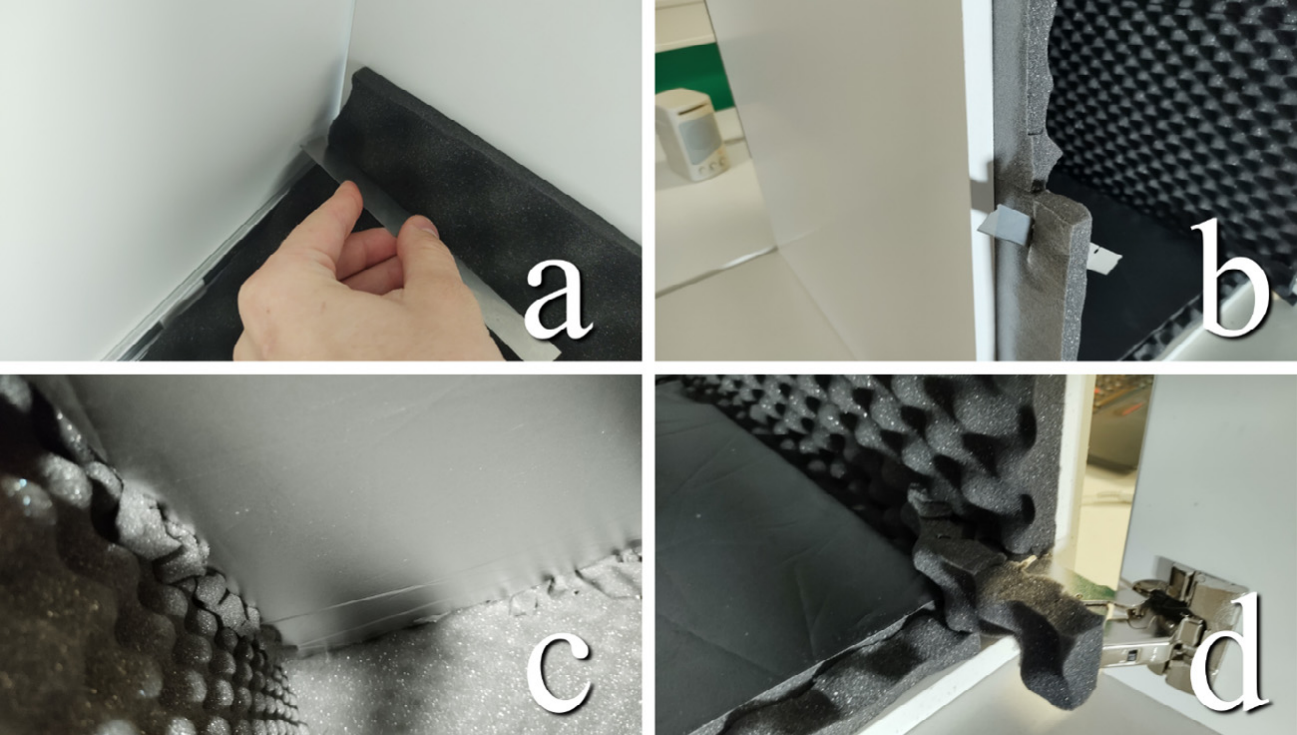
a) A sharp knife is used to cut excess foam in the back corner. b) excess foam is trimmed away at the front. c) Where the foiled panels meet the convoluted foam, cut-offs can be added to remove any gaps. d) Cut away foam to allow the hinges to move.

The sheets of foam are precoated with adhesive with which they can be attached and fitted in the chamber, as seen in Fig. 12. The “egg carton” shaped foam panels (“Acoustic foam”) are used along the walls, starting on the back. A sharp knife is used to trim away excess foam, making sure that there is a tight interconnection and overlay between the panels where they meet. The foiled acoustic foam panels are used on the top and bottom to reflect light as well as provide flat surfaces to mount lights on and work on. Cut-offs are used to fill gaps between the foiled and convoluted panels, as well as cover the door.

#### Add Magnet door closer

**This step requires the following:** Magnet(2×), CA glue, Utility knife, Drill driver with screw bits

**Fig. 13:**
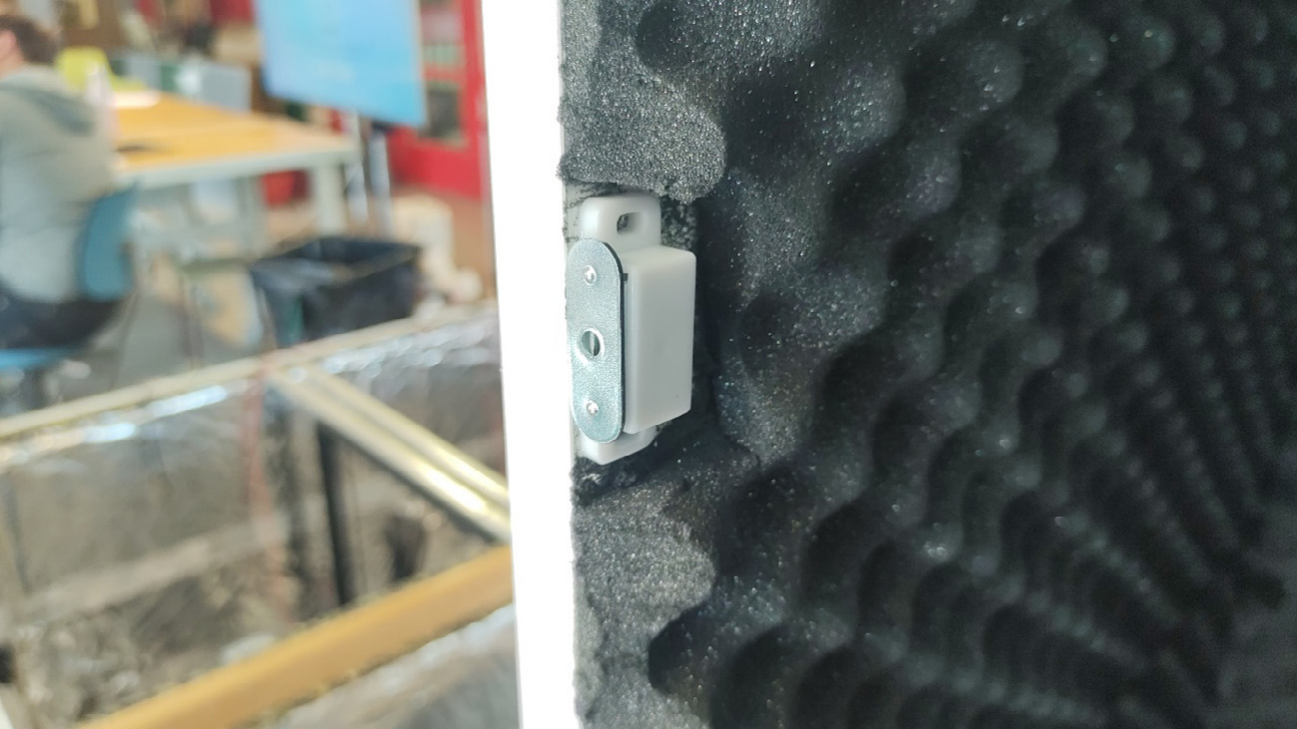
Cut away foam to fasten the magnet.

With the added foam, it might be necessary to add magnets to help the door stay shut. A simple magnetic door closer can be mounted by cutting away some of the foam, as seen in Fig. 13. Use CA glue to align and fasten the pieces. Add screws to secure them.

#### Drill holes for eyebolts

**This step requires the following:** Concrete_Table.dxf, MDF, Laser cutter, Pen, Drill driver with drill bits, Measuring tape

**Fig. 14:**
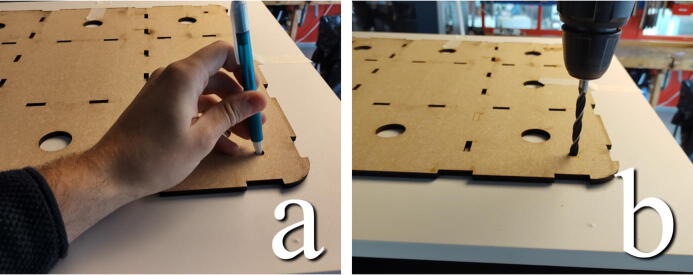
a) Mark hole pattern. b) Or use the holes to guide the drill bit.

In this step, the file Concrete_Table.dxf should be cut from 6 mm MDF. The plate with the larger diameter round holes is used to mark out holes for the eyebolts that connect the cabinet and table through springs. Place the plate on top of the cabinet and measure an equal distance between all the sides of the plate and the edges of the chamber. Make a mark at the center of each of the 8 mm corner holes, as seen in Fig. 14. The plate can also be used to get the holes started by clamping it down and using an 8 mm drill bit directly in the holes. Drill all the way through the top board and foam.

#### Aluminum loadbearing beams

**This step requires the following:** Aluminum profiles, Hacksaw, Measuring tape, Drill driver with drill bits

**Fig. 15:**
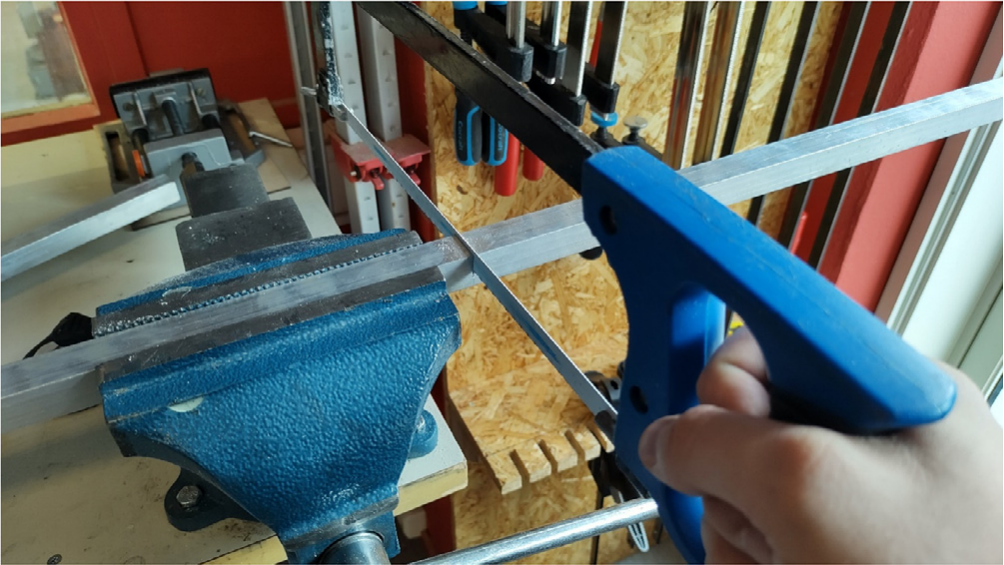
Saw the aluminum profile before drilling holes in it.

To distribute the weight of the table into the walls of the cabinet, two aluminum bars are added to the top of the cabinet. The bars are cut 600 mm long (Fig. 15). Use the plate from the previous step to mark out two holes on each bar with the same spacing as in the previous step. The holes are drilled with an 8 mm drill bit.

#### Gasket and eyebolts

**This step requires the following:** Aluminum profiles (previous step), Gasket material, Eyebolts or hooks, Drill driver with drill bits

Eyebolts or hooks are added to later attach the table to through springs. Though eyebolts are used in the presented build, hooks may be a better choice when available as it will mitigate the need for further fastening mechanisms between the eyebolts and springs. Which, in turn, reduces the part count and frees up space for further deformation of the springs. Before mounting the eyebolts, a rubber gasket should be added underneath the aluminum bar to mitigate the transfer of vibrations. Use a sharp knife to rough cut strips ∼ 15–20 mm wide so that they fit underneath the aluminum bars. Test fit the gaskets under the bar, use a drill to puncture the gasket so that the eyebolt is able to pass through. Remove more material around the hole with a utility knife if needed.

**Fig. 16:**
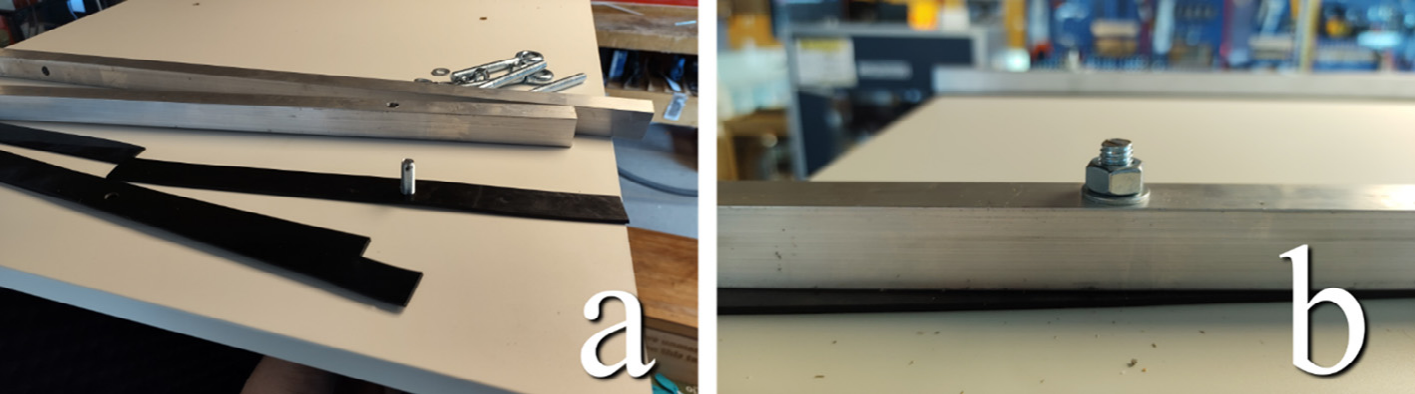
a) Gasket material is cut in strips and punctured to allow eyebolts to pass through. b) Gasket, aluminum bar, eyebolt, washer, and nut fully assembled.

Mount the eyebolts from the inside of the chamber, through the gasket and the aluminum bar, add washer and nut on top, and hand-tighten them. The nut should be screwed on so that the bolts stick out of the top, as seen in Fig. 16. Any further tightening can be done later as a way to fine adjust leveling of the table.

### Table build

Next, the table is built. The primary purpose of the table is to provide a high mass and a flat surface upon which equipment can be placed. The design proposed utilizes a laser cutter to make a mold in which a concrete table is cast. However, multiple methods can be utilized to achieve a high mass flat surface. Such as cutting and drilling a suitable metal plate or filling a wooden box with concrete or sand.

#### Gather mold parts

**This step requires the following:** MDF parts cut from Concrete_Table.dxf

Gather the parts from “Concrete_Table.dxf” cut previously. The proposed table measures 450×450×62mm with holes for the eyebolts spaced 370 mm center to center. If a laser cutter is not available, a similar dimensioned box design can be cut using hand tools into which concrete can be poured.

#### Assemble inner structure

**This step requires the following:** MDF parts cut from Concrete_Table.dxf, Wood glue

**Fig. 17:**
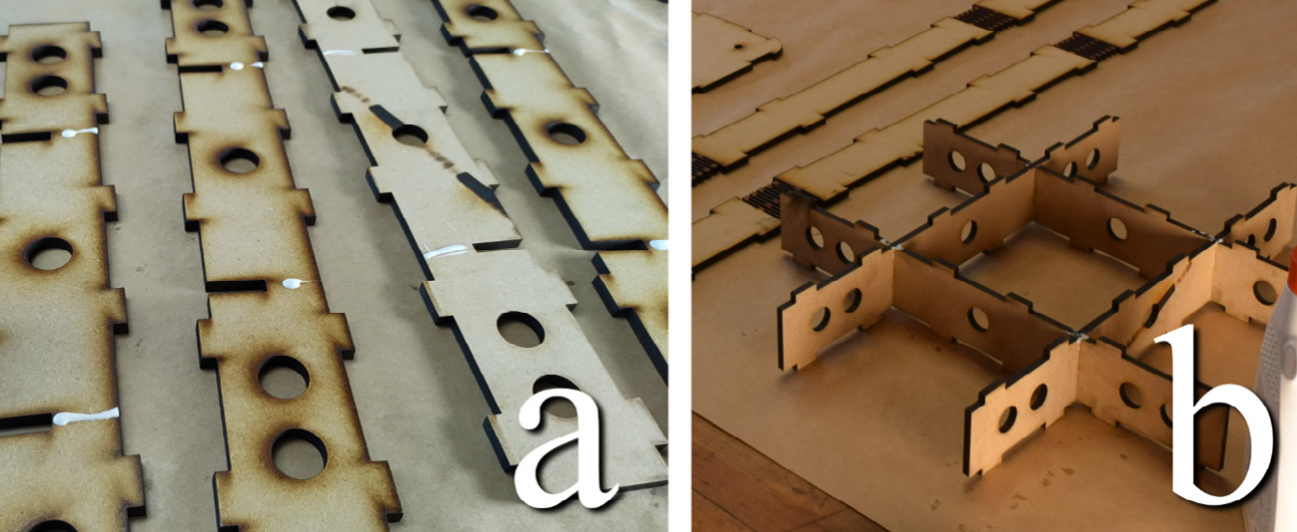
a) Glue along slits. b) Puzzle together.

The thinner straight pieces in the “Concrete_Table.dxf “ file interlock to form an inner structure in the table to align the top and bottom. Add glue along the slits of the pieces and puzzle them together, as seen in Fig. 17.

#### Glue the inner structure to the top plate

**This step requires the following:** MDF parts cut from Concrete_Table.dxf, Wood glue, Hammer

**Fig. 18:**
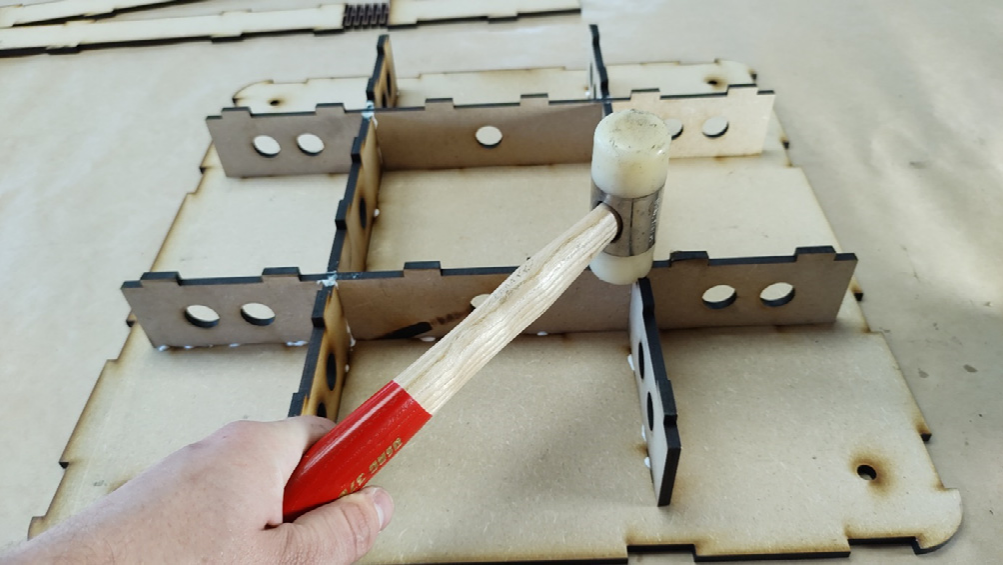
Use a hammer to force the inner structure together with the top plate.

Add glue along the meeting edges and finger joints of the inner structure from the previous step. The structure should interlock with the top plate of the concrete table, the larger piece without the larger diameter holes in it, as seen in Fig. 18. A small mallet can be used to force it into its receiving holes.

#### Add the exterior walls

**This step requires the following:** MDF parts cut from Concrete_Table.dxf, Wood glue, Masking tape

**Fig. 19:**
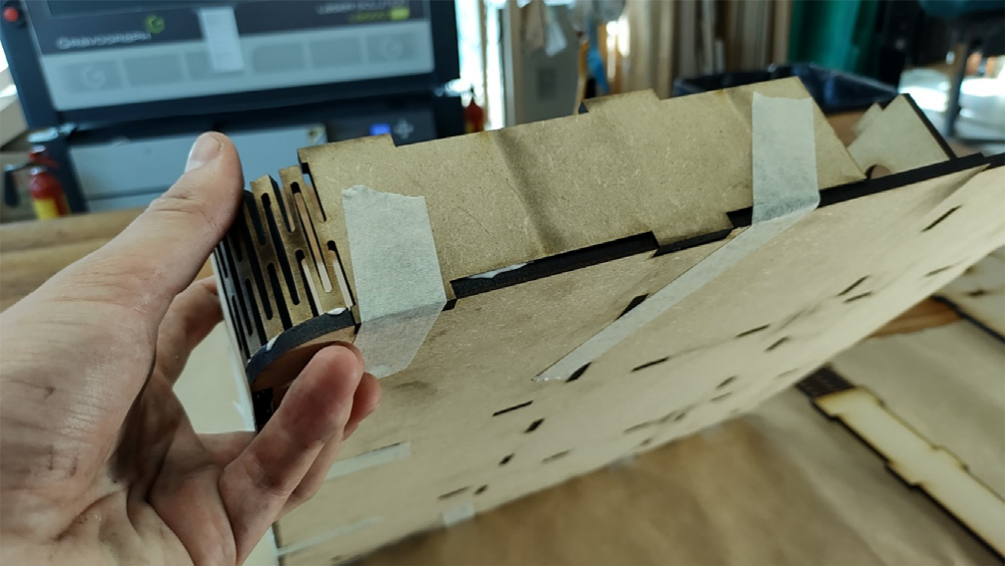
Use masking tape to hold glued pieces together.

The exterior walls of the table are glued along the edges, using the finger joints to align it with the top plate. Take special care with the curved living hinge corners, ensuring that they run flush with the plate. Masking tape can be used to hold everything in place while the glue sets, as seen in Fig. 19.

#### Align and fix pieces

**This step requires the following:** CA glue, CA glue accelerator

**Fig. 20:**
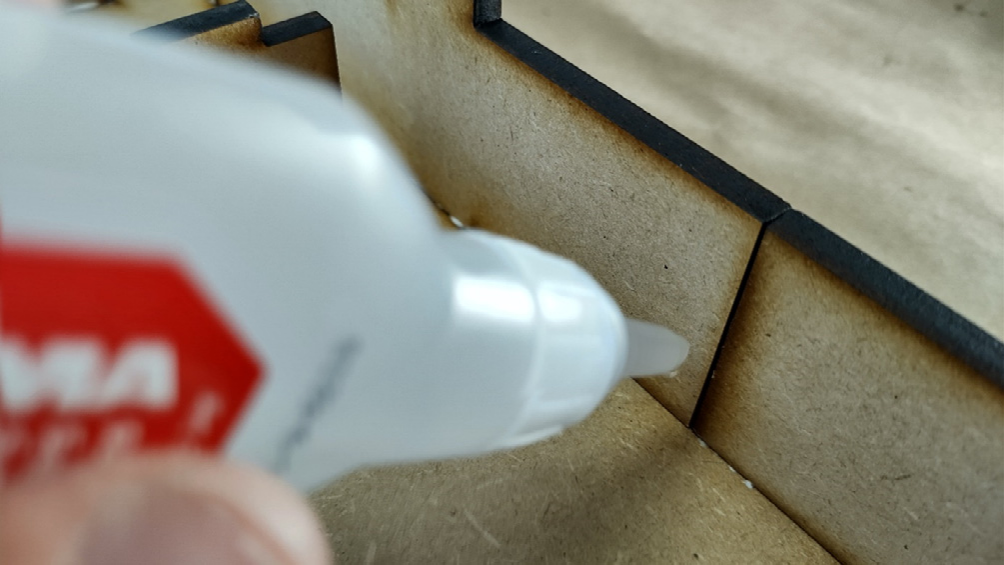
Add CA glue to fix tricky alignments quickly.

Where the two exterior walls meet (Fig. 20), it can be beneficial to apply a dab of CA glue, align the pieces by hand, and fix them into position with an accelerator. The same trick can be used to ensure that the edge of the curved corners stays flush with the curve of the plate.

#### Install eyebolts in mold

**This step requires the following:** 4×Eyebolts or hooks, Putty

**Fig. 21:**
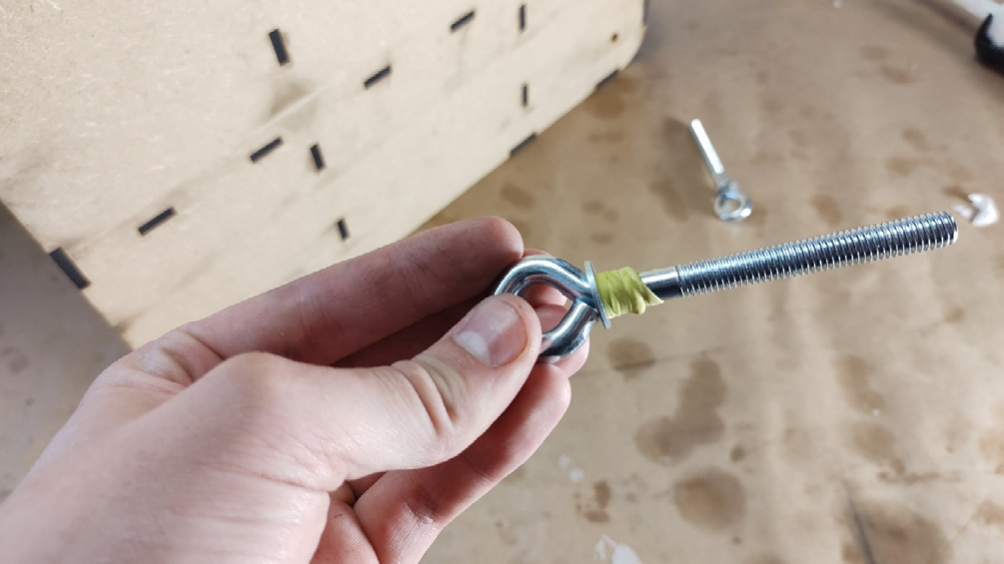
Gasket made of vacuum bagging sealing tape.

The eyebolts or hooks should now be installed in the mold. Add a washer and push it all the way to the top. Make a casket out of a putty (Fig. 21), such as vacuum bag sealing tape or plasticine, so that the eyebolt/hook will completely plug up its receiving hole once installed.

#### Final preparations before casting

**This step requires the following:** Metal wire, Duct tape

**Fig. 22:**
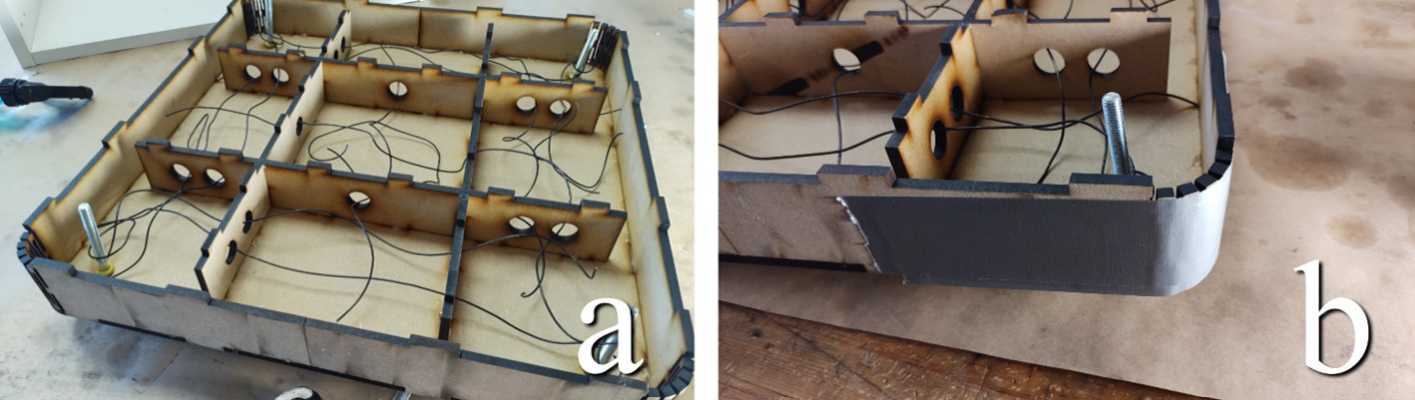
a) Add metal wires in the mold. b) Cover corners and gaps with duct tape.

The inner structure is made so that it should easily be able to receive metal wires for a simple arming of the concrete, add wires as seen in Fig. 22a. Add duct tape to the rounded corners of the mold to seal them, as seen in Fig. 22b. If glued well, the edges of the mold should not leak, but duct tape can be added along the edges as a precaution. Make sure that the mold is positioned level on the eyebolt heads and that the eyebolts are in the wanted rotational position.

#### Mix and Pour concrete

**This step requires the following:** Concrete mix, Drill driver, Bucket, Concrete stirrer drill attachment, MDF “bottom plate” cut from Concrete_Table.dxf

**Fig. 23:**
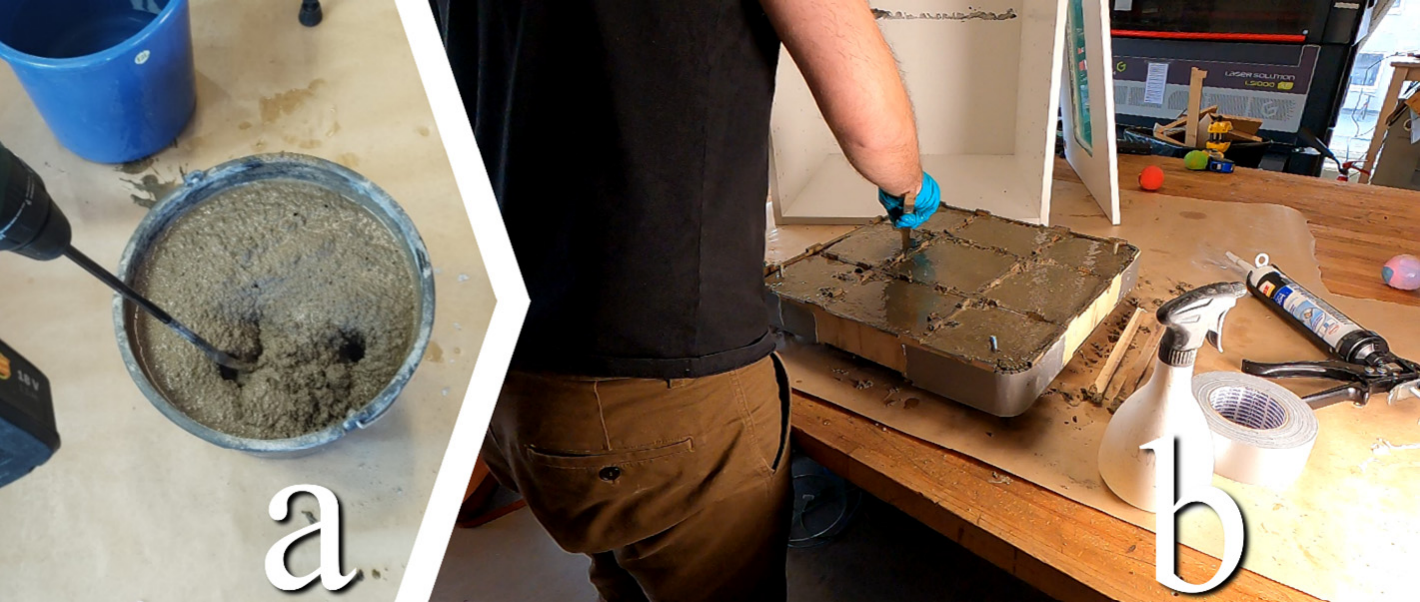
a) Mix concrete thoroughly in accordance with package. b) Pour concrete, distribute and pack it with a stick.

Mix and stir the concrete mix in a bucket in accordance with the ratios on the package (Fig. 23a). Pour the concrete into the open mold, working section by section. Use a small stick to shake and work the concrete into all of the areas of the mold and eventually level the concrete (Fig. 23b). Once the mold is evenly filled, add the final MDF piece: the bottom plate. The MDF might have swelled end deformed some at this point. Try wiggling the pieces together while exerting some force. Clamps may be used to try to force the pieces together. Make sure that the eyebolts enter their holes on the bottom piece. Once installed, fix it by adding washers and nuts on the eyebolts.

#### Add felt (optional)

**This step requires the following:** Felt, Spray adhesive, Utility knife

**Fig. 24:**
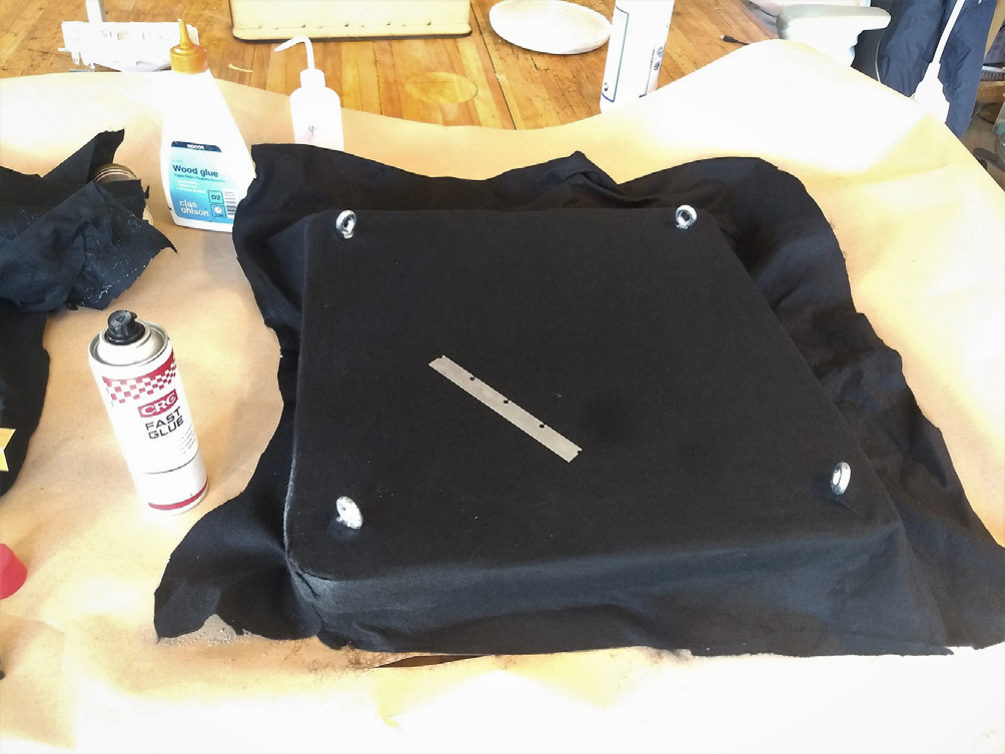
Felt added to the top of the table.

We decided to add felt to the surface of the table, both to generate higher friction in the interaction with smaller equipment placed on the table and to reduce the number of hard surfaces with regard to the acoustic reflections within the chamber. The felt is fixed to the table with spray adhesive. Holes for the eyebolts are roughly cut before applying the glue to both the felt and the table. When the glue has gone tacky, the felt can be worked onto the table by pressing it down and working creases out to the edges, as seen in Fig. 24. A sharp knife is used to cut the corners and work the felt down the sides. Cut the excess felt in the corners and along the bottom skirt. Add another flat sheet to the bottom.

### Springs, Dampers, and final assembly

Finally, the spring and damper system can be made, connecting the table with the chamber.

#### Create coiled springs

**This step requires the following:** Spring steel wire, Spring winding tool, Pliers

The most critical and challenging part of this build is the design and manufacturing of the metal springs. To achieve and test the appropriate characteristics, we made the springs ourselves. The below-described dimensions are the result of testing different designs. The final springs have a K value of approximately 0.57 N/mm, which was derived by applying a known load and measuring the deformation of a spring.

**Fig. 25:**
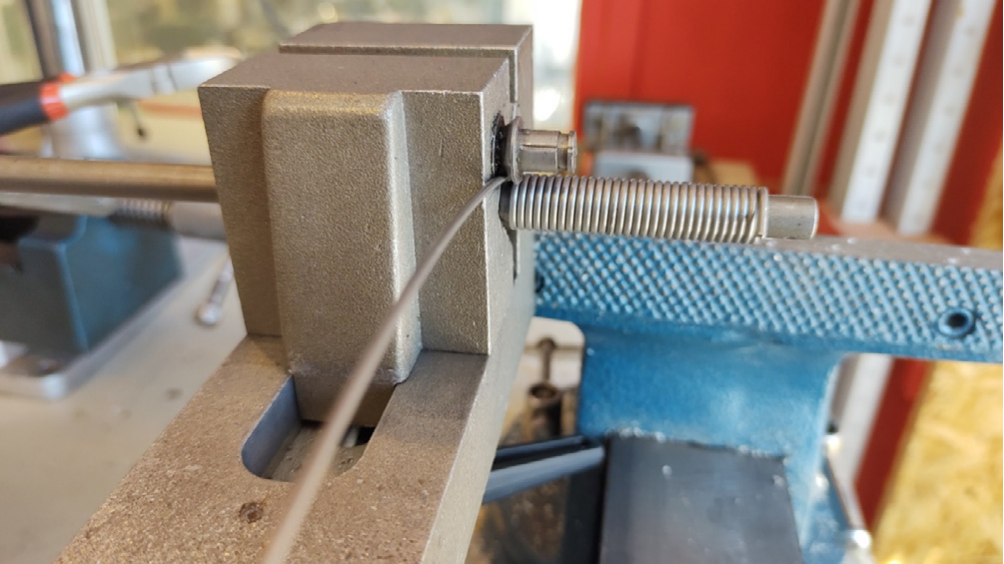
Spring steel winded in spring winding tool.

The springs used were hand-coiled by twisting a 1.5 mm spring steel wire around a 7 mm metal rod in a variable pitch spring winding tool (the pitch is set to zero and follows the diameter of the wire), as seen in Fig. 25. With the spring back, the resulting inner diameter is measured to be 9 mm. The spring is coiled approximately 90 times. To ensure that the springs have an appropriate length, the springs are later plastically deformed to their final dimensions once installed in the chamber. For the weights used in our chamber, the springs final lengths in their relaxed state are 280 mm.

#### Secure the end loops of the springs

**This step requires the following:** Solder, Solder Flux, Soldering Iron

**Fig. 26:**
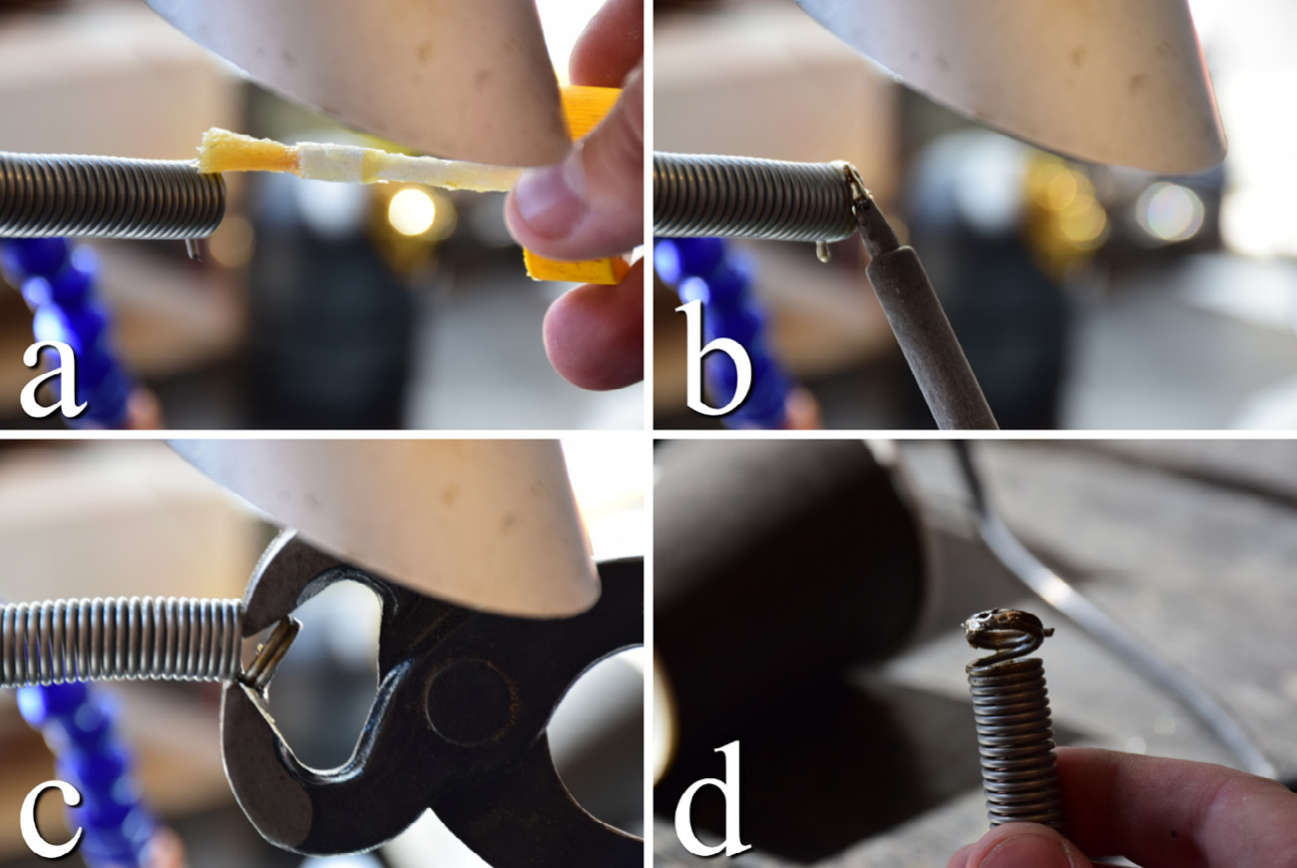
a) Apply flux. b) Solder the top two coils. c) Use pliers to pinch out the connector hoop. d) Finished connection point.

During installation, the springs will be stretched to their final dimensions and will operate close to their plastic region. As a precaution, it is advisable to form and secure the ends of the spring where it connects to the chamber and platform. For this purpose, we found it to be sufficient to solder the ends using 40/60 led/tin solder. Add flux to the two top coils of spring. Let the tip of a warm soldering iron (∼450degrees Celsius) rest between the two coils, add generous amounts of solder. Keep applying heat until a good wetting and bond have been achieved. Once cooled, pliers can be used to angle the connected coils up for easier connection. The process is illustrated in Fig. 26.

#### Attach springs to the roof of chamber

**This step requires the following:** Zip Ties

**Fig. 27:**
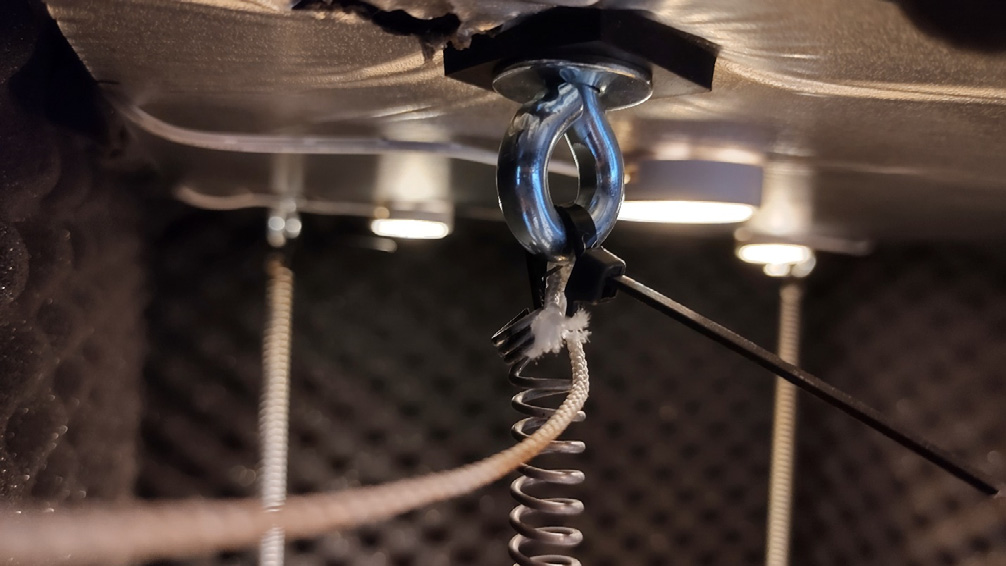
Springs connected to eyebolts with zip ties.

The springs can now be installed in the chamber. To decrease the space used by the springs and thus maximize the length along which the springs can elongate, the top connection should take up little space. This is achieved by using robust zip ties, as seen in Fig. 27. Run zip ties through the eyes of the eyebolts and the springs connecting all four springs to a corresponding eyebolt in the roof of the chamber. If hooks were used in step 4.1.12 and 4.2.6, the springs may be connected directly.

#### Install table in chamber

**This step requires the following:** Zip ties (or carabiners)

**Fig. 28:**
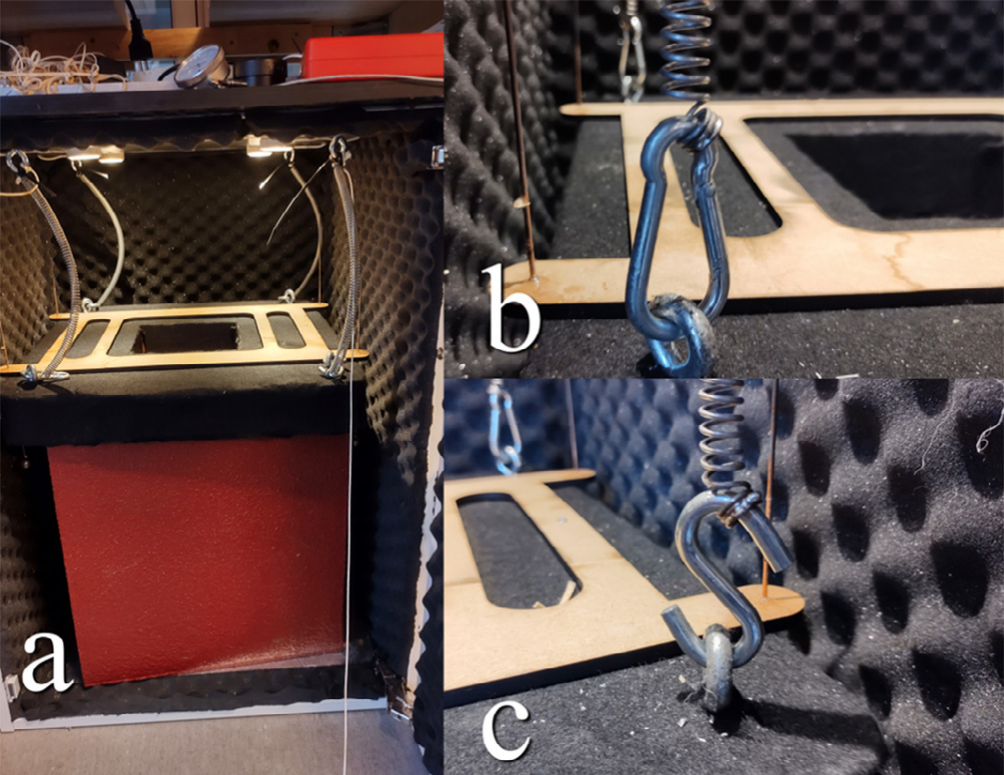
a) Raise the table on something while installing it. b) Carabiners is a reversible alternative to zip ties. c) S-hooks may also be used.

It is now time to install the table in the chamber as illustrated in Fig. 28. With help, the table should be lifted into the chamber. Raise the table from the floor of the chamber by adding support such as solid plastic boxes or wooden blocks underneath it. The table should be high enough that the clearance between it and the roof of the chamber is close to the length of the springs. The springs can now be attached to the eyebolts on the table, connecting the springs directly above to the eyebolts directly below. For a reversible connection, it can be wise to use either s-hooks or carabiners, although zip ties can be used here as well. If hooks were used in step 4.1.12 and 4.2.6, the springs may be connected directly.

#### Remove support and lower table

Carefully remove the support under the table while lifting the table. Lower the table until it is supported by the springs alone. The table should have a generous clearance to the bottom (>30 cm) at this point.

#### Prepare material for dampers

**This step requires the following:** Damper_Sphere_Fastening, Dampers_Baseplate.dxf, MDF

The table is dampened by suspending four steel spheres in a high viscosity fluid. The four spheres are attached to an MDF plate that is screwed onto the table. The pools into which the spheres are suspended are in turn connected to a bottom plate that rests on the floor of the chamber. The two plates can be laser cut from 6 mm MDF. Alternatively, the included dxf can be used as a template to rough cut a similar shape.

#### Damper fluid reservoirs

**This step requires the following:** Acrylic glass, Acrylic_Damper_Tubs.dxf, Acrylic glue, masking tape, Laser cutter

**Fig. 29:**
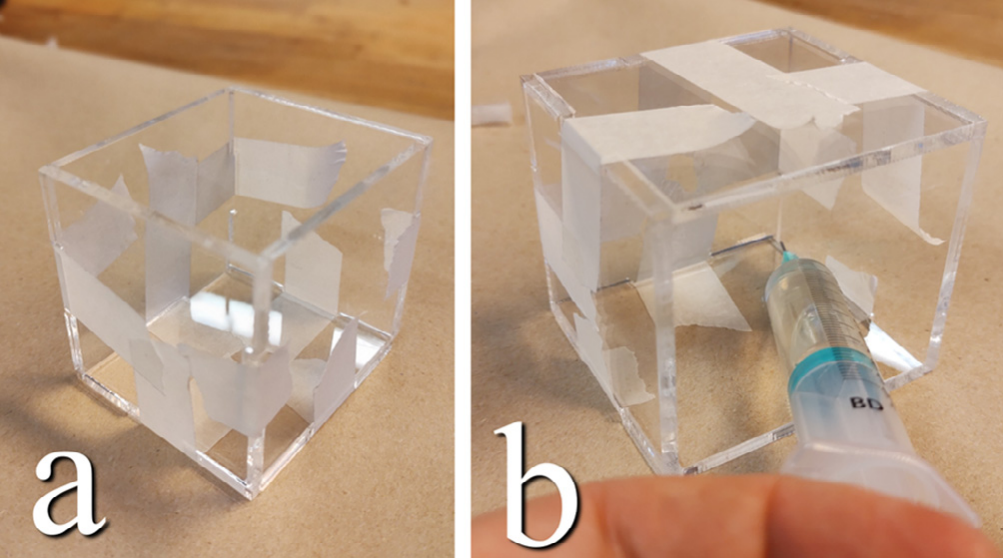
a) Assemble acrylic pieces with tape. b) Apply acrylic glue along inner edges with syringe.

Included are files to cut out four containers into which the high viscosity fluid is contained. The containers can be cut in 3 mm acrylic sheets and glued using appropriate glue (Fig. 29). The walls of the containers are assembled with masking tape. Apply glue along the inside edges with a syringe. Alternatively, one could find four small plastic containers of a similar size to use, such as empty food containers or cups.

#### Install damper reservoirs at the bottom of the chamber

**This step requires the following:** Honey, Dampers_Baseplate.dxf, CA glue (or double-sided tape)

**Fig. 30:**
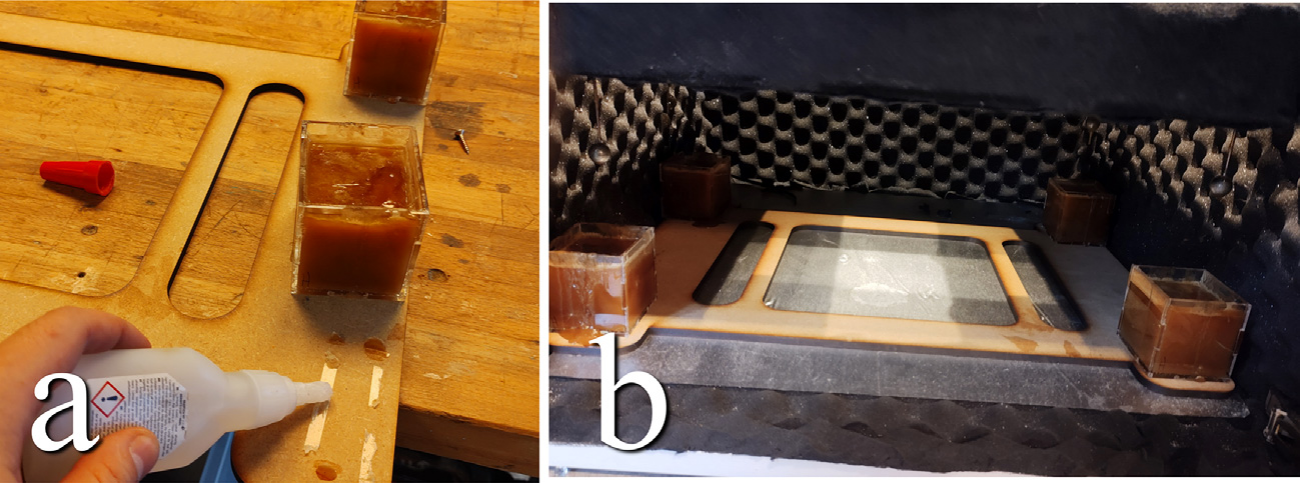
a) Glue the filled containers to the outer corners of the plate. b) The plate fits snugly in the bottom of the chamber.

The fluid containers can now be filled. As the dampener fluid, we propose to use honey, as it is generally possible to find with varying, but high, viscosities [17], [18] and is far cheaper than e.g. silicone oils. Fill the containers with honey and place them in their corners on the bottom plate. We attached the containers using double-sided tape or small dabs of CA glue (Fig. 30).

#### Solder damper spheres

**This step requires the following:** Welding rod, 4× Bearing spheres, Solder, Solder Flux, Soldering Iron, Multitool with metal grinding disk

**Fig. 31:**
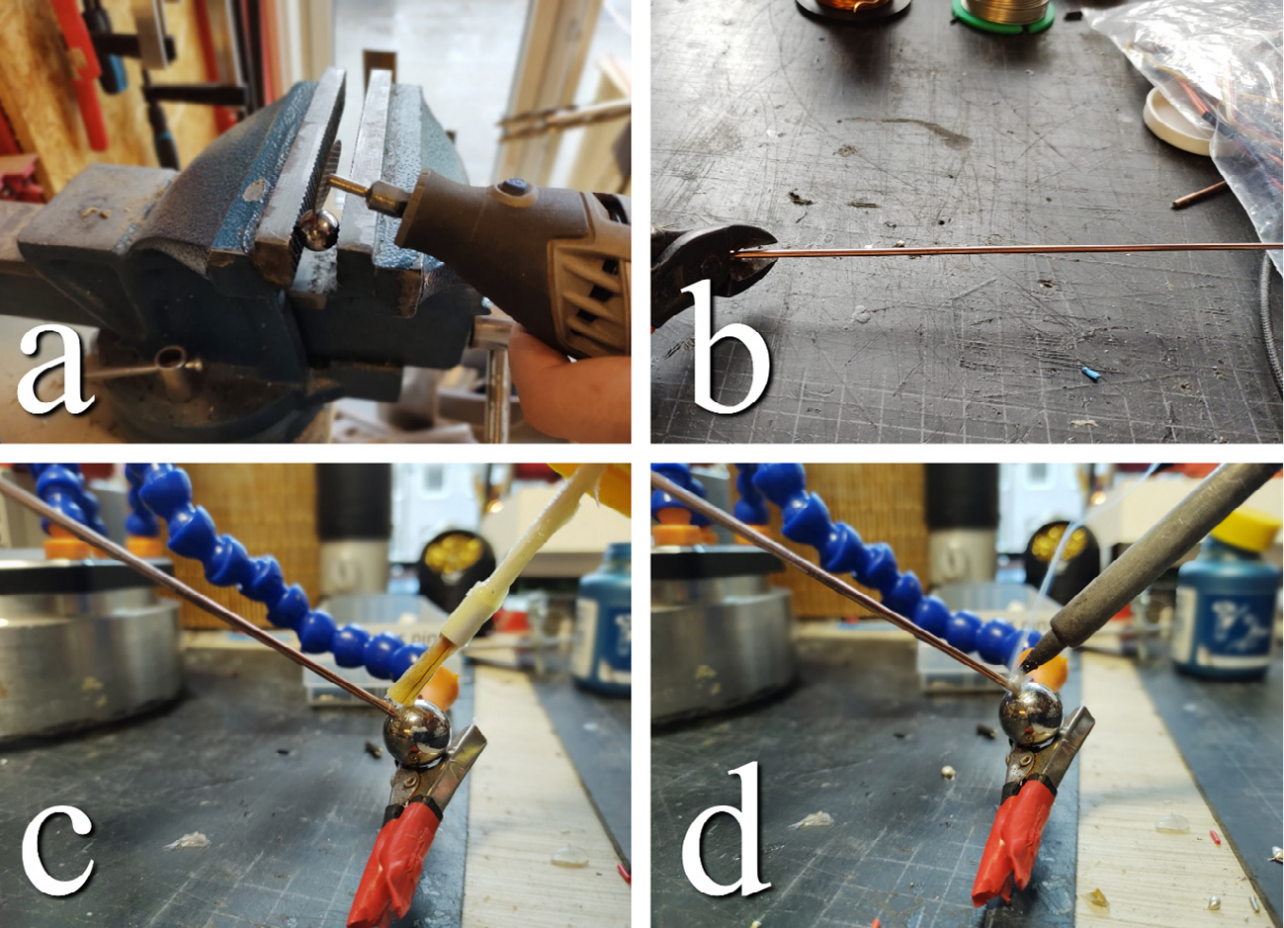
a) Grind flat surface on sphere. b) Cut welding rods to length. c) Align rod and sphere and apply flux. d) Solder.

The ball-bearing spheres will act as dampers suspended in the high viscosity fluid. The spheres need to be connected to the table. This can be achieved by attaching a rod to the spheres. Using a grinding wheel on a multitool, gently make a small flat surface on the spheres. Cut welding rods to approximately 30 cm lengths. Use clamps, “helping-hands”, or tape to fix the sphere and welding rod so that the rod end meets the flat surface of the spheres. Add flux and solder the two together. The process is illustrated in Fig. 31. If available, threaded rods and steel spheres with threaded receiving holes could be a suitable alternative to the presented design.

#### Install spheres in plate

**This step requires the following:** CA glue

**Fig. 32:**
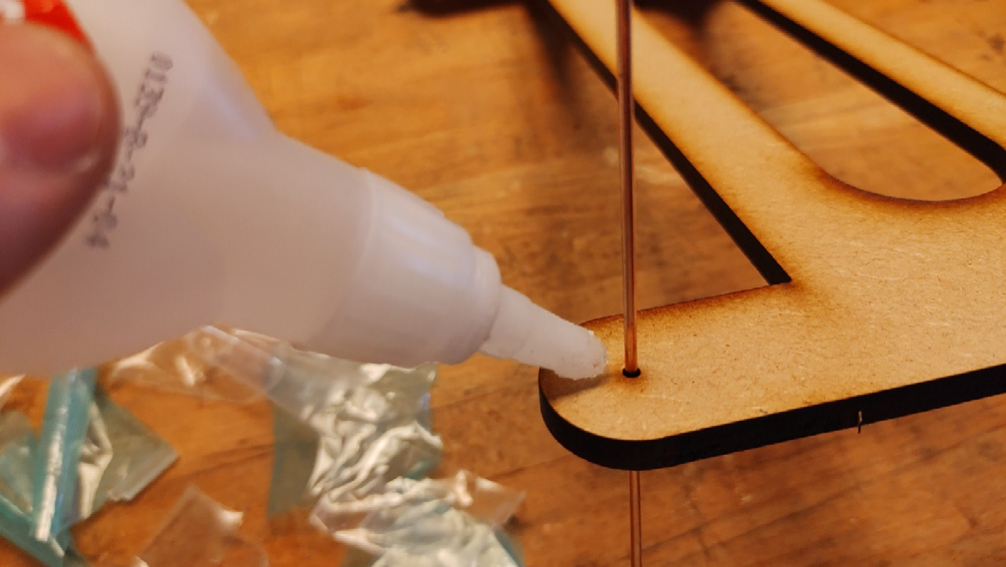
Insert damper rods into plate and apply CA glue.

The spheres are installed by forcing the rods through the holes of their receiving plate. Add a small dab of CA glue to prevent it from slipping (Fig. 32). Adjust this height later by breaking the glue bond with pliers.

#### Attach Bearing plate to table

**This step requires the following:** 2× Wooden screws, Drill driver with drill bits

**Fig. 33:**
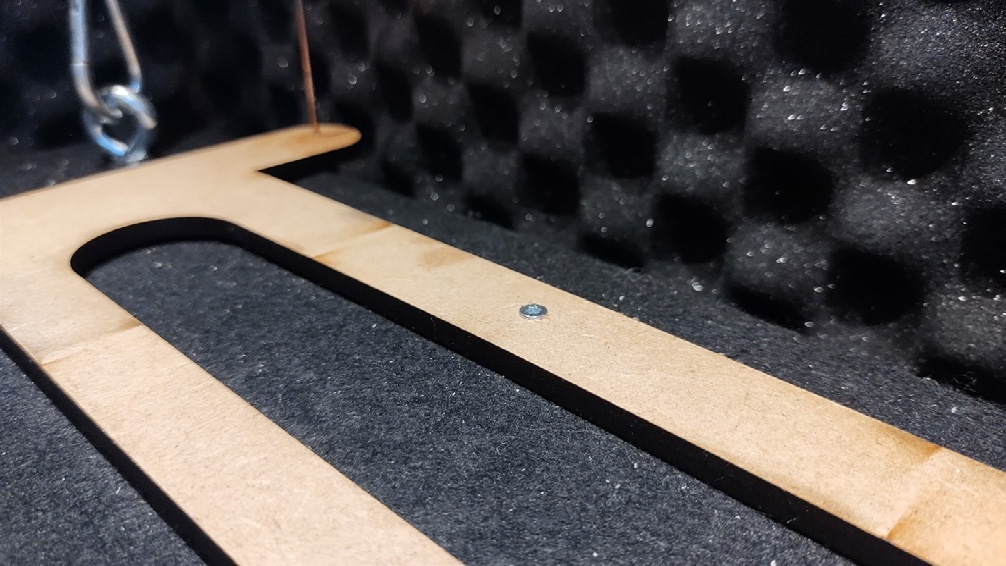
The damper plate can be screwed directly onto the table.

The plate with the attached spheres on rods is put on top of the table with the spheres facing down. Two screws attach the plate and the table (Fig. 33).

#### Lower table to operating height

**Fig. 34:**
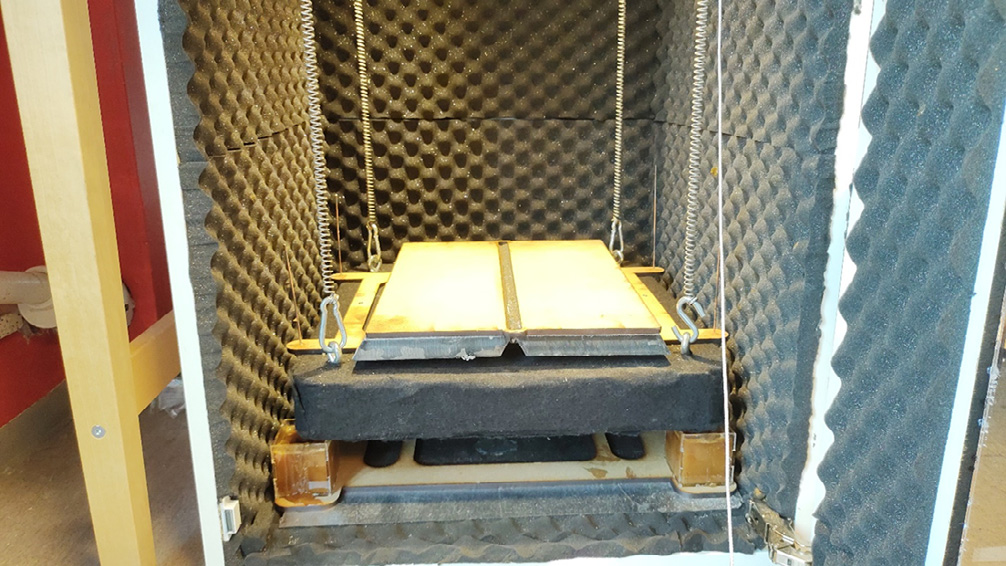
Table in its final operating heigh with steel plates as weights.

The table is lowered by first increasing the weight of the table to the maximum operating weight. Depending on the weight and spring parameters, there might still be clearance between the spheres and dampening reservoirs. To remove this gap, the table is pushed down to plastically deform the springs until the table is only a few centimeters above the damper fluid reservoirs. Adjust the metal sphere rod lengths so that the spheres are fully submerged in the honey. The final height is shown in Fig. 34.

#### Add lights (Optional)

**This step requires the following:** Lights, Drill driver with drill bits

**Fig. 35:**
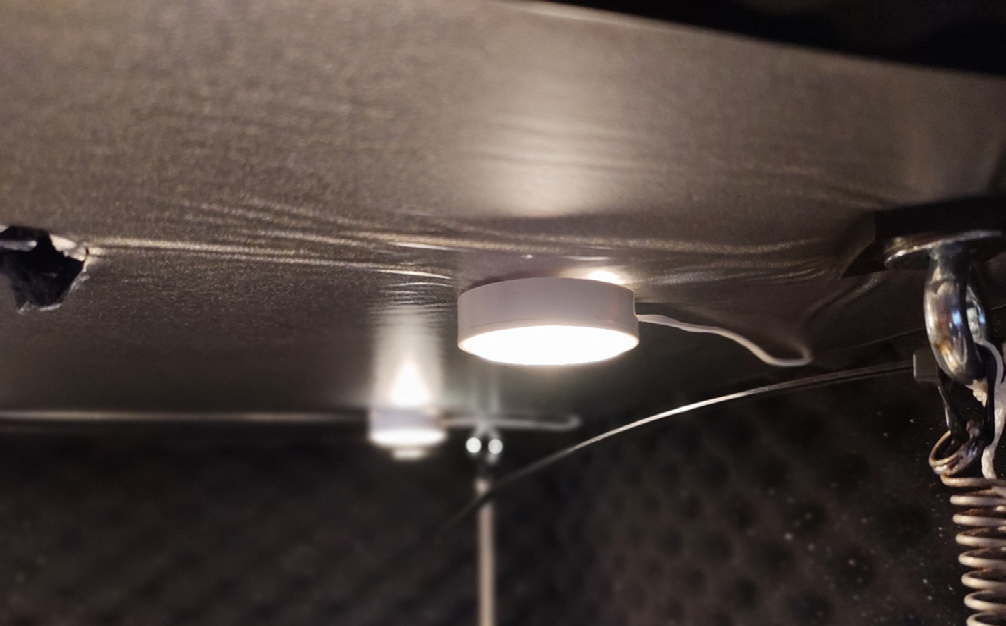
The lights are attached to the roof of the table with the included double-sided tape.

Lights can be added to the roof of the chamber by attaching them with included double-sided tape. Run the wires out the back of the chamber by drilling holes (Fig. 35).

#### Install chamber on rubber gaskets

**This step requires the following:** Gasket material

When placing the chamber in its final destination, it can be heightened and leveled by placing rubber gasket sheets under its four corners.

#### Add Elastic bands to springs

**This step requires the following:** Elastic bands

**Fig. 36:**
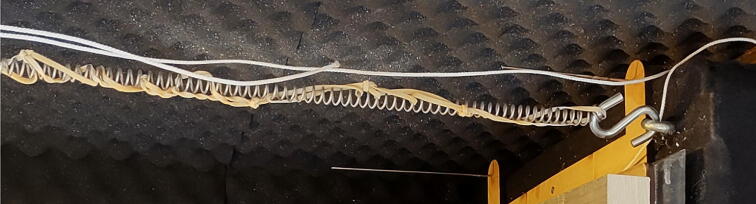
Elastic bands tie together to dampen steel spring resonance.

The internal resonance of the steel springs can be simply mitigated by loosely twisting elastic bands into and around the springs [11], as seen in Fig. 36. For lower frequencies we noticed little change in the systems performance, yet it is a simple precaution to implement.

## Operation instructions

**Fig. 37:**
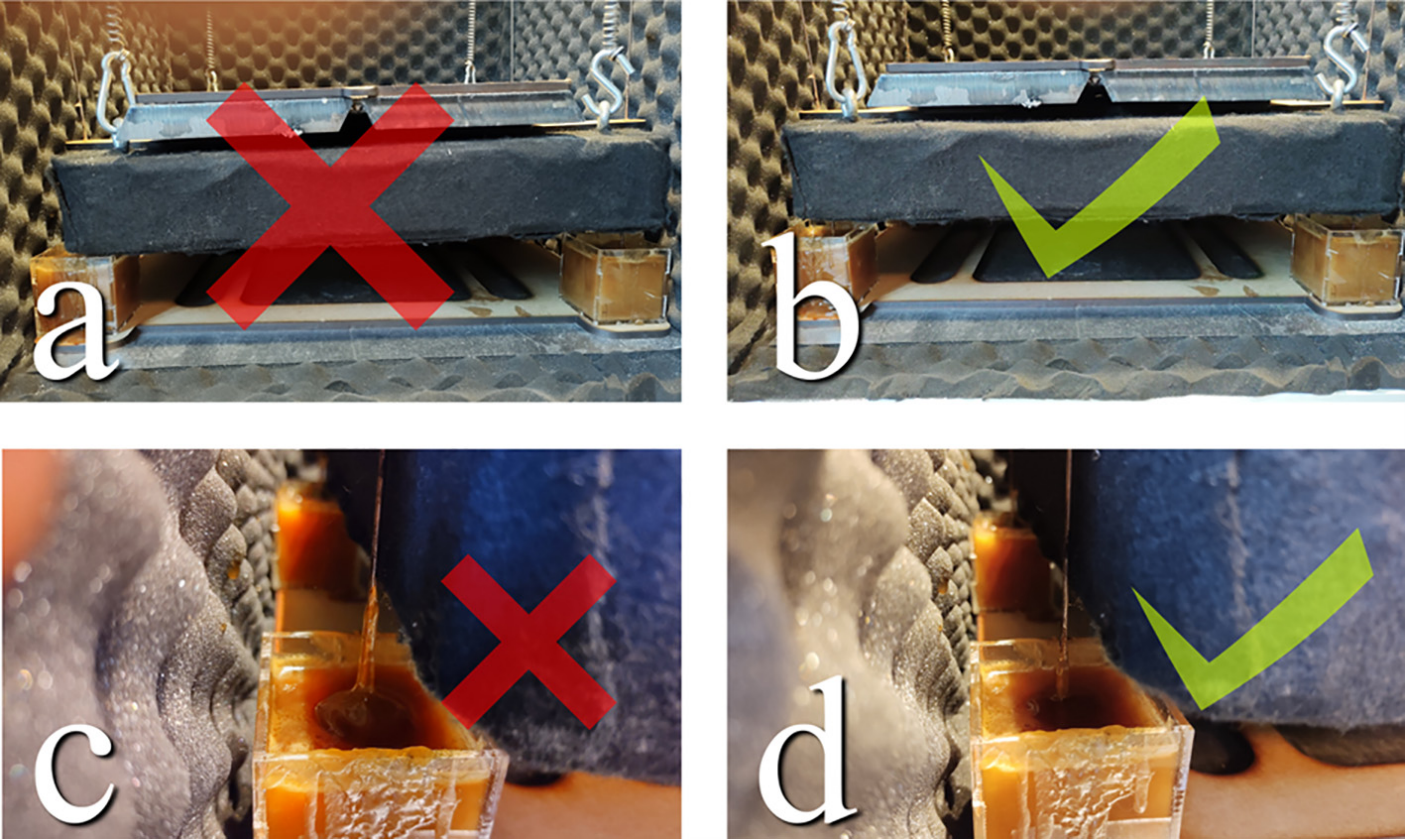
The weight is not evenly distributed, and the table is crooked. Additionally, the table touches the damper reservoir. b) Table is leveled. c) Table is too high up and the spheres are not fully submerged in the dampening fluid. d) Spheres are fully submerged.

Equipment and experiment setups sensitive to vibrations are placed and integrated on the table in the chamber to isolate them from external vibrations. The weight and placement of the equipment should be considered and balanced so that the steel spheres are evenly submerged in the dampening fluid but do not touch the bottom or walls (Fig. 37). To level the table and achieve an appropriate operating height, we add steel plates as weights. In addition, the rods of the ball bearings may be slid up and down and re-glued, though, for consistent performance, it is recommended to keep the table at a similar height for all experiments. To run power and signal wires for experiments, holes can be drilled anywhere along the chamber walls and roof.

### Safety

The metal springs may fail if they are subjected to excessive forces, this could potentially hurt the operator. Safety glasses should be worn when working in the chamber. The connection points of the springs should also be inspected prior to use. If there are signs of deformations or cracks in the soldering, install new springs or repair the damage.

### Resonance and temperature control

The spring-damper design proposed will resonate for frequencies at approximately 1.2 Hz, which may coincide with walking and human movements. It is recommended to place the chamber in an area with low foot traffic and to reduce movement around the chamber when sensitive experiments are in progress. Further, due to the addition of foam to the walls, energy sources within the chamber may cause the chamber temperature to rise. For this reason, it is recommended to keep power supplies and larger electronics outside the chamber and run needed signals and power by wire to the experiment equipment. Additionally, though the lights in the chamber have low power consumption, we recommend not having them turned on continuously for the above-mentioned reasons.

## Validation and characterization

**Fig. 38:**
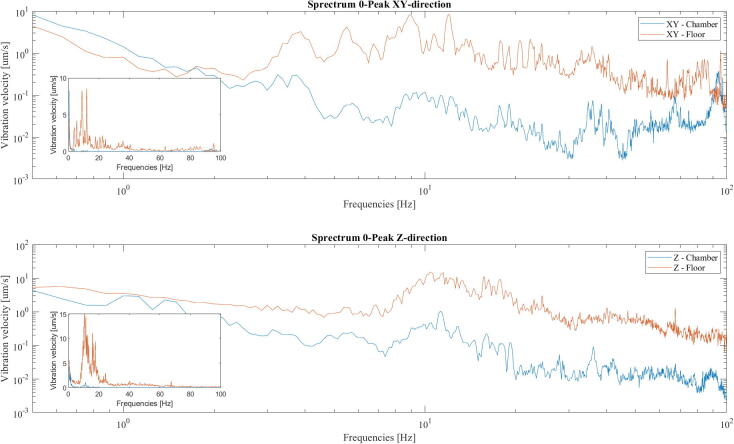
Log-log plot of the accelerometer data in the vibration isolated chambered against the accelerometer data on the floor. The vibrations are significantly reduced both in the vertical and the horizontal directions. Inset contains the same data plotted to a linear scale.

To measure the performance of the vibration isolation in the chamber a Wilcoxon 731A (Wilcoxon Sensing Technologies, USA) accelerometer unit and a SC11 Compact Analysis System (Spicer Consulting Limited, England) was used. The accelerometer measures along a single axis between 0.1 and 500 Hz. The primary culprit of vibrations in the range 5–20 Hz in the lab where the chamber is located is due to local traffic, and more specifically, two bus routes operated by longer hinged busses. Due to this, the vibrations were sampled as successive spectrums, combined to show the maximum 0 to peak measurement for each frequency in the data acquisition period. This way, it could be made sure that vibrations caused by the passing of both busses would be reflected in the spectrum, with little significance regarding the number of passes and amount of traffic. As both bus routes operate at a frequency of 7–10 min, data was sampled over 1000 s to ensure that a bus of each type would pass in the timeframe. The successive spectrum is derived from 125 samples of 800 data points sampled in the range 0–100 Hz with a Hanning window applied. The accelerometer was placed upright on the floor and on the table in the chamber, denoted as the Z-direction in Fig. 38. Further, the sensor was placed on its side, denoted as the XY-direction in Fig. 38. The amplitudes of the vibrations are significantly reduced on the vibration isolated table as compared to the floor. When considering the transmissibility of the presented system, we would expect there to be an increase in amplitudes around the resonance frequencies of the table, though for the measurements in the horizontal direction there is an apparent increase in vibrations around and lower than the resonance frequency, the same can not be seen in the data sampled in the vertical direction. This may be due to the system being subjected to differing vibrations in this frequency range during the sampling periods, as the data was not sampled in parallel. As such, is not necessarily illustrative of the general transmissibility of the design. The presented test was primarily designed to contain information on the assumed culprits of vibrations in the 5–20 Hz range, and successfully show an obvious reduction in vibrations in this range.

**Fig. 39:**
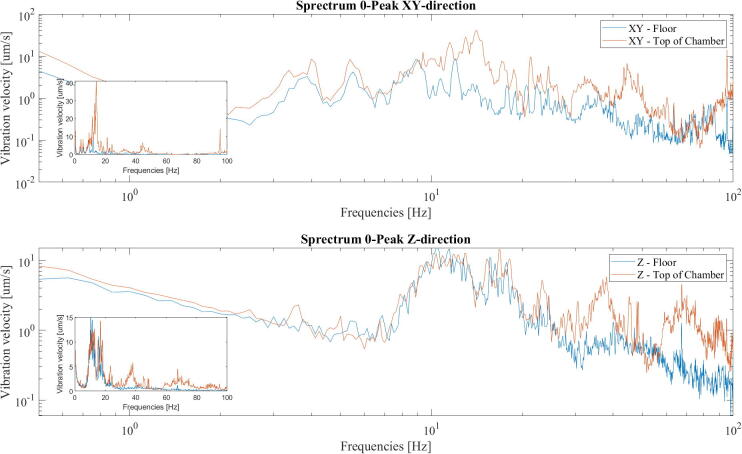
Log- log plot of accelerometer data from the top plate of the chamber compared against the floor. Inset is plotted in linear scale.

Further, a test was made by placing the accelerometer on the roof of the chamber to give an idea of the stability of the structure. The accelerometer was placed right above the door, where the lateral stability of the chamber is the lowest. The results can be seen in Fig. 39. We see that for the vertical measurements, the vibrations are similar on top of the chamber and the floor in the critical 5–20 Hz range, with apparent amplifications for higher frequencies above 20 Hz. In the horizontal direction, the vibrations are significantly amplified in the critical 5–20 Hz range. This was expected and is something that can be mitigated by further reinforcing the structure in future iterations. In addition, it might be helpful to investigate the influence of other materials as feet to further isolate the chamber from the floor. Where pieces of rubber gaskets are now used, rubber tubes [12] or tennis balls may also be viable alternatives in reducing the transmissability of vibrations, in the interesting frequency ranges, between the floor and the chamber.

### Resonance and lower frequencies

The chamber drastically reduces the vibrations in the critical range 5–20 Hz, yet due to its dimensions and simple design, it will experience resonance at approximately 1.2 Hz. These lower frequencies will typically resonate with walking, which can be troublesome in areas with excessive foot traffic. This is not necessarily reflected in the vibration data presented previously, as it was sampled in periods of little foot traffic for the sake of consistency between the samples. Mitigating the influence of walking around the chamber can be done by making the chamber taller, if its placement allows it, as the resonance frequencies of the springs are linked to their length of deformation. In further iterations, implementing multiple stages and introducing concepts of non-linear vibration isolation though alternative spring configurations, may be possible to reduce the dynamic stiffness and resonance frequency of the system [14], [19].

### Sound

**Fig. 40:**
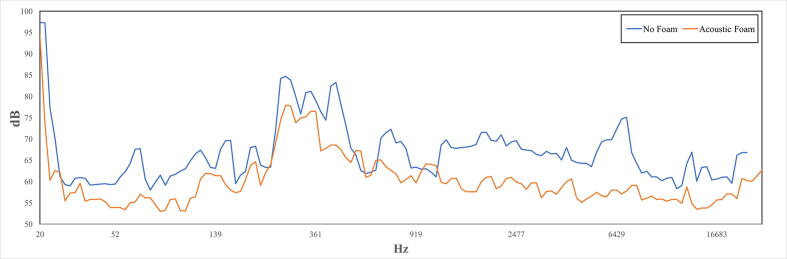
Sound level measurements inside the chamber before and after installation of polyurethane foam, frequencies are approximately matched by timestamp and initial knock.

During the installation of the acoustic polyurethane foam, a simple test was made to measure the changes in sound penetration into the chamber. A set of speakers were placed outside the chamber a sound sequence was played ranging from 20 Hz to 20 kHz [20] at a set volume. Data was logged with a SL-4023SD (Lutron Electronics, Inc., USA) sound level meter before the installation of foam and after. The sampled data was synchronized to the sound sequence frequencies by knocking on the chamber when the sequence was started, to show the approximate frequency response in Fig. 40. There is a noticeable reduction in the sound levels in the chamber after the installation of the foam, especially for the higher frequencies from around 1000 Hz, which is expected from pure polyurethane foams [21]. The sound level meter was set to sample with minimal bias, and the uneven volume levels for different frequencies is most likely due to the speakers’ uneven frequency response. The authors would like to note that the chamber is not explicitly designed for sound insulation and absorption, and better solutions may be possible with little extra effort. However, for a quick improvement in sound insulation and absorption, evidently, the self-adhering acoustic foam panels can be easily installed on the large surfaces inside the chamber.

### Use

**Fig. 41:**
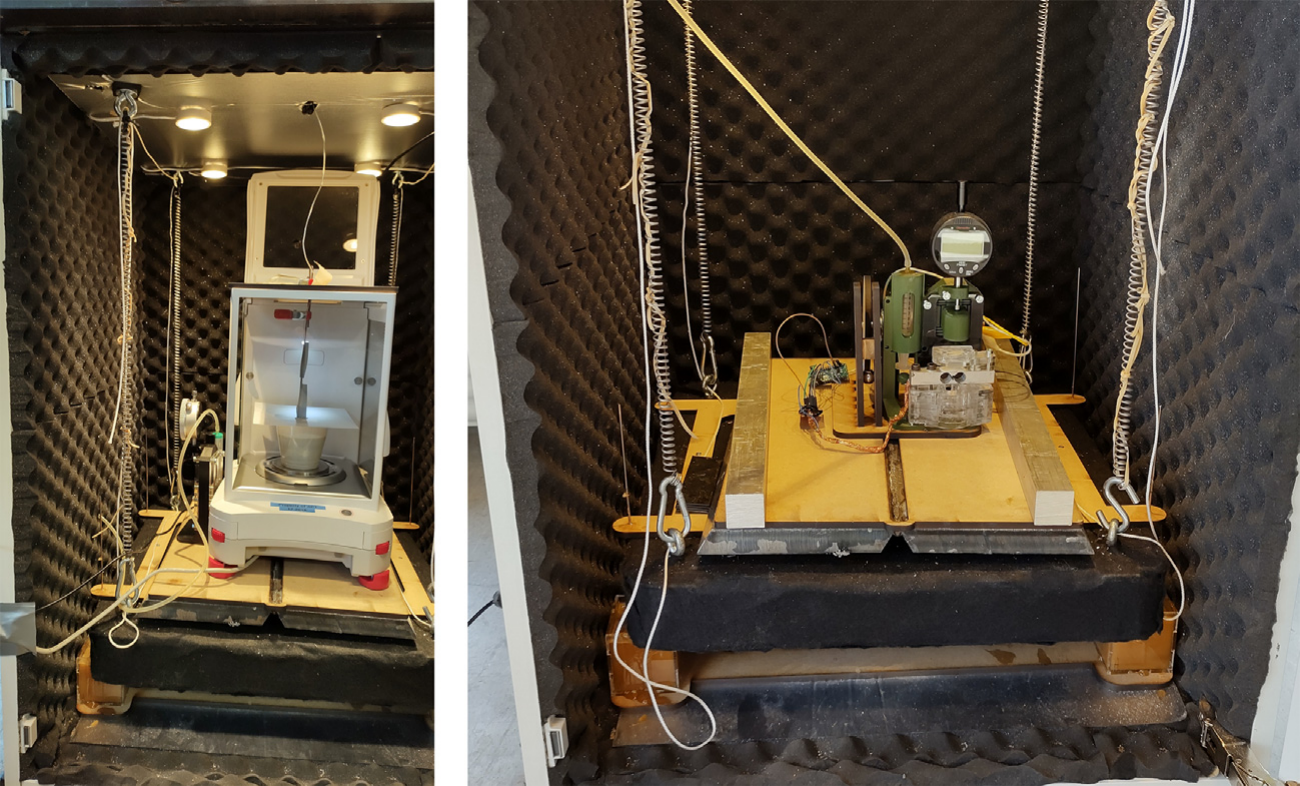
To the left, a precision balance is placed in the chamber. To the right, equipment for measuring piezoresistive response of a sensor material are placed in the chamber.

**Fig. 42:**
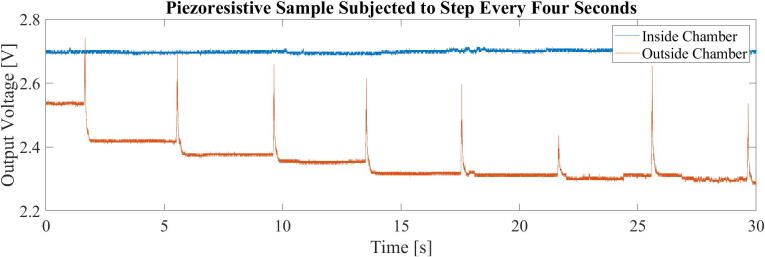
The output voltage over a voltage divider including a piezoresistive material sample changes when subjected to vibrations due to a heavy stomp on the floor, approximately 1 m from the sample. A voltage change is not visibly obvious in the sample inside the chamber.

The chamber has been used to enable further measurements of the response of piezoresistive carbon fiber silicone composites to enable their characterization for use in soft robotics solutions (Fig. 41). To demonstrate the chambers practical significance when dealing with said composites, the experiment in chapter 1, Fig. 1, was repeated with a sample placed on the vibration isolated table. A comparison between the sample placed on the floor and a sample placed on the table is presented in Fig. 42. Though some smaller peaks are still evident in the data from the vibration isolated composite sample, their amplitude is far lower, and their frequency is not obviously relatable to the induced vibrations. The reduced influence of vibrations will further enable controlled investigation into the behavior of these piezoresistive materials.

The chamber has also been used to reduce vibrations on a precision balance when measuring the delivery of small insulin units, where the viscous nature of the insulin solution resulted in inconsistent readings when no vibration isolation precautions were taken. Both projects are pictured in Figs. 41. The simple construction means that projects can easily and quickly use the chamber and make alterations such as drilling holes for wires and tubes when necessary. It is our opinion that a simple vibration isolation chamber, such as the presented design, is a valuable addition to any research and product development laboratories where sensitive experiments and prototype tests are carried out.

## Declaration of Competing Interest

The authors declare that they have no known competing financial interests or personal relationships that could have appeared to influence the work reported in this paper.
